# In Chronic Lymphocytic Leukemia the JAK2/STAT3 Pathway Is Constitutively Activated and Its Inhibition Leads to CLL Cell Death Unaffected by the Protective Bone Marrow Microenvironment

**DOI:** 10.3390/cancers11121939

**Published:** 2019-12-04

**Authors:** Filippo Severin, Federica Frezzato, Andrea Visentin, Veronica Martini, Valentina Trimarco, Samuela Carraro, Elena Tibaldi, Anna Maria Brunati, Francesco Piazza, Gianpietro Semenzato, Monica Facco, Livio Trentin

**Affiliations:** 1 Department of Medicine-DIMED, Hematology and Clinical Immunology Unit, University of Padua, 35128 Padua, Italy; filippo.severin@unipd.it (F.S.); federica.frezzato@unipd.it (F.F.); andrea.visentin@aopd.veneto.it (A.V.);; 2 Veneto Institute of Molecular Medicine, 35128 Padua, Italy; 3 Department of Molecular Medicine–DMM, University of Padua, 35128 Padua, Italy

**Keywords:** chronic lymphocytic leukemia, JAK2 inhibitor, STAT3 inhibitor, microenvironment, mesenchymal stromal cells, ibrutinib

## Abstract

The bone marrow microenvironment promotes proliferation and drug resistance in chronic lymphocytic leukemia (CLL). Although ibrutinib is active in CLL, it is rarely able to clear leukemic cells protected by bone marrow mesenchymal stromal cells (BMSCs) within the marrow niche. We investigated the modulation of JAK2/STAT3 pathway in CLL by BMSCs and its targeting with AG490 (JAK2 inhibitor) or Stattic (STAT3 inhibitor). B cells collected from controls and CLL patients, were treated with medium alone, ibrutinib, JAK/Signal Transducer and Activator of Transcription (STAT) inhibitors, or both drugs, in the presence of absence of BMSCs. JAK2/STAT3 axis was evaluated by western blotting, flow cytometry, and confocal microscopy. We demonstrated that STAT3 was phosphorylated in Tyr705 in the majority of CLL patients at basal condition, and increased following co-cultures with BMSCs or IL-6. Treatment with AG490, but not Stattic, caused STAT3 and Lyn dephosphorylation, through re-activation of SHP-1, and triggered CLL apoptosis even when leukemic cells were cultured on BMSC layers. Moreover, while BMSCs hamper ibrutinib activity, the combination of ibrutinib+JAK/STAT inhibitors increase ibrutinib-mediated leukemic cell death, bypassing the pro-survival stimuli derived from BMSCs. We herein provide evidence that JAK2/STAT3 signaling might play a key role in the regulation of CLL-BMSC interactions and its inhibition enhances ibrutinib, counteracting the bone marrow niche.

## 1. Introduction

JAK/Signal Transducer and Activator of Transcription (STAT) signaling is one of the most investigated and important pathways involved in tumorigenesis and cancer sustainment. Four different Janus Family Kinases (JAKs) and the seven downstream members of the STATs protein family, which combine as homo- and heterodimers to exert their transcriptional activity upon their phosphorylation at tyrosine residues, transduce signals triggered by more than 40 cytokines [1]. Deregulation of JAK/STAT signaling pathways, leading to inappropriate activation of STATs, has been recognized in several hematological malignancies as a critical factor supporting survival, proliferation and metabolism of neoplastic cells [2,3,4,5]. The JAK2/STAT3 in particular has a key role in conveying signals that elicit the transcription of pro-survival and anti-apoptotic genes, such as Mcl-1 and Bcl-2, both known to be over-expressed in chronic lymphocytic leukemia (CLL) [6,7].

CLL is characterized by mature clonal B lymphocytes that proliferate within secondary lymphoid organs, such as bone marrow, lymph nodes, spleen, therefrom invading the peripheral blood. The survival of the neoplastic clone is supported by intrinsic defects of CLL cells, such as the activation of the B-cell receptor (BCR), the overexpression and activation of the Src kinase Lyn [8,9], Bruton tyrosine kinase (BTK) [10] and Bcl-2 [11] proteins, and extrinsic microenvironmental factors derived from the bone marrow or lymph nodes [12]. We and other groups previously demonstrated that bone marrow mesenchymal stromal cells (BMSCs), the most represented stromal cells within the bone marrow, support the survival as well as the drug resistance of CLL cells through cell-cell contacts and soluble molecules such as CXCL10, CXCL12 [13], and interleukin 6 (IL-6), the main activator of JAK2/STAT3 signaling [14]. While Jak2-mediated STAT3 phosphorylation on tyrosine (Tyr) 705 is known to promote dimerization and subsequent localization to the nucleus, the phosphorylation on serine (Ser) 727, which is promoted by several protein kinases, Erk1/2 and Cyclin-dependent kinases among others, seems to be critical for optimal STAT3 function as a transcription factor, facilitating its activity in cooperation with other transcriptional cofactors including AP-1 and p300 [15]. Conversely, in CLL STAT3 was found constitutively phosphorylated at Ser727 with respect to normal B lymphocytes [16], but the regulation of the phosphorylation at Tyr705 deserves further investigation.

Ibrutinib, the first in-class BTK inhibitor, showed a broad activity in B-cell hematological malignancies, such as CLL, in either treatment naive or relapsed-refractory patients [17]. Ibrutinib can induce a high and prolong rate of overall responses in CLL, though not completely eradicating neoplastic cells within the bone marrow niche [18]. This observation led us to speculate that BMSCs, through the modulation of JAK2/STAT3 pathway, could be implicated in CLL cell survival and ibrutinib-resistance. Given the importance of the JAK2/STAT3 axis for neoplastic cell survival, the investigation of this pathway and its regulation could improve our understanding of CLL pathophysiology and might help to identify additional signaling networks of potential therapeutic interest.

We herein demonstrated that in CLL cells both JAK2 and STAT3 are over-expressed as compared to normal B lymphocytes, STAT3 is constitutively phosphorylated on Tyr705 in the majority of patients, and bone marrow-derived MSCs sustain CLL survival through the activation of JAK2/STAT3 signaling. Moreover, the targeting of JAK2/STAT3 triggers CLL cell time- and dose-dependent apoptosis, the inhibition of Lyn through the re-activation of the phosphatase SHP-1, and enhances ibrutinib activity partially overcoming BMSC support. These data provide new insights into the pathogenesis of CLL focusing the attention on tumor microenvironment and the JAK2/STAT3 pathway.

## 2. Results

### 2.1. JAK2/STAT3 Axis Is Over-Expressed and Constitutively Activated in CLL Neoplastic B Cells

**Figure 1:**
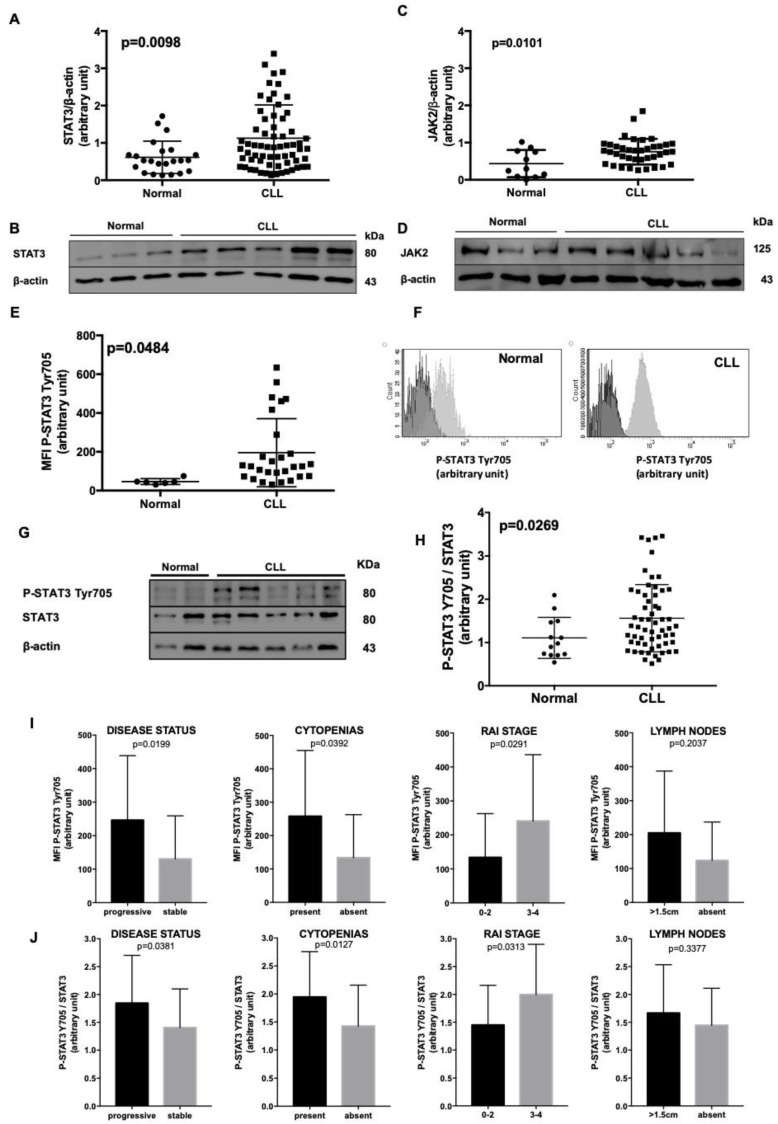
STAT3 characterization in normal B lymphocytes and CLL cells. (**A**), (**C**). Densitometry of STAT3/β-actin (**A**) and JAK2/β-actin (**C**) ratio in age-matched healthy subjects vs. CLL patients. (**B**), (**D**). These panels show a representative blot of 3 normal controls and 5 CLL patients. STAT3 and JAK2 expression is higher in CLL samples with respect to healthy donors. (**E**) Flow cytometry evaluation of STAT3 phosphorylation at Tyr705. The MFI of P-STAT3 on Tyr 705 was higher in CLL (N = 25) than normal B lymphocytes (N = 6). (**F**) Representative histograms for the evaluation of P-STAT3 Tyr705 MFI, comparing it with the Fluorescence Minus One (FMO), in normal and CLL cells (dark grey: FMO; light grey: MFI). (**G**) This panel shows a representative blot of 2 normal controls and 6 CLL patients. At basal condition STAT3 was phosphorylated on Tyr705 in most CLL cells as compared to healthy controls. Neoplastic cells show a heterogeneous expression pattern, see also Figure S3B. (**H**) Densitometry of P-STAT3 Tyr705/STAT3 ratio of normal subjects (n = 13) vs. CLL patients (n = 59). At basal condition P-STAT3 Tyr705/STAT3 ratio is higher in CLL than normal B lymphocytes, even with some differences among patients (Mann–Whitney test, *p* = 0.0269). (**I**) Correlation between STAT3 Tyr705 phosphorylation assessed by flow cytometry and clinical parameters. We correlated P-STAT3 Tyr 705 MFI levels from 37 patients with clinical variables. We found a significant higher STAT3 phosphorylation on Tyr705 in progressive (*p* = 0.0199), cytopenic (*p* = 0.0392) patients with a more advanced Rai Stage (*p* = 0.0291). Although patients with enlarged lymph nodes show a median MFI higher than subject without adenopathy, this difference was not statistically significant (*p* = 0.2037). (**J**) We correlated P-STAT3 Tyr 705/STAT3 ratio levels from 59 patients with clinical variables. We found a significant higher STAT3 phosphorylation on Tyr705 in progressive (*p* = 0.0381), cytopenic (*p* = 0.0127) patients with a more advance Rai Stage (*p* = 0.0313). Although patients with enlarged lymph nodes show a median level higher than subjects without adenopathy, this difference was not statistically significant (*p* = 0.3377). Cytopenia was defined as hemoglobin <100 g/L, absolute neutrophil count <1000/µL, platelets <100,000/µL. MFI = Median Fluorescence Intensity; FMO = Fluorescence Minus One.

By flow cytometry and Western Blotting (WB) analysis, we assessed STAT3 protein levels in freshly isolated neoplastic B cells obtained from 66 untreated CLL patients and in normal B lymphocytes from 23 age-matched healthy subjects. We found significant higher levels of both STAT3 (1.13 ± 0.11 vs. 0.61 ± 0.09; Mann–Whitney test, *p* = 0.0098, Figure 1A,B) and JAK2 (0.76 ± 0.06 vs. 0.44 ± 0.11, Mann–Whitney test, *p* = 0.0101, Figure 1C,D) proteins in CLL cells as compared to normal B lymphocytes. We also assessed the mRNA levels of Jak2 and Stat3 in normal and CLL cells, as previously described [19]. Although no significant differences emerged for Jak2 mRNA, we found a higher amount of Stat3 mRNA in CLL cells with respect to normal B lymphocytes (Figure S1). Subsequently, we correlated STAT3 and JAK2 expression with the most relevant biological prognostic markers (e.g., cytogenetics abnormalities [20], Immunoglobulin Heavy Chain Variable region (IGHV) mutational status [21], and Integrated CLL Scoring System (ICSS score) [22], Figure S2), but no statistical differences were highlighted, suggesting that both proteins are homogeneously over-expressed in CLL patients. 

By flow cytometry (Figure 1E,F) and WB (Figure 1G), we evaluated the phosphorylation status of STAT3 in CLL cells. According to data coming from the literature, in all CLL cases STAT3 was phosphorylated on Ser727 at basal condition (Figure S3A). Interestingly, we also found a constitutive STAT3 phosphorylation on Tyr705 as compared to normal B lymphocytes, in 86% of patients (Median Fluorescence Intensity (MFI): Normal cells, N = 6: 46.50 ± 6.12 vs. CLL cells, N = 25: 211.3 ± 35.85; Mann–Whitney test, *p* = 0.484, WB: Normal cells, N = 13: 1.11 ± 0.13 vs. CLL cells, N = 59: 1.56 ± 0.10; Mann–Whitney test, *p* = 0.0269, Figure 1G,H). We correlated P-STAT3 Tyr705 MFI and protein levels from CLL patients with clinical variables (Figure 1I,J). We found a significant higher MFI of P-STAT3 Tyr705 in progressive (*p* = 0.0199 and *p* = 0.0381), cytopenic (*p* = 0.0392 and *p* = 0.0127) patients with a more advanced Rai Stage (*p* = 0.0291 and *p* = 0.0313). Although patients with enlarged lymph nodes show a median MFI higher than subjects without adenopathy, this difference was not statistically significant (*p* = 0.2037 and *p* = 0.3377) (Figure 1I,J). Moreover, we found a positive correlation between the MFI levels of STAT3 Ser727 and STAT3 Tyr705 phosphorylation in 23 patients analyzed by flow cytometry (Pearson correlation, *p* = 0.0003; Figure S3C).

**Figure 2:**
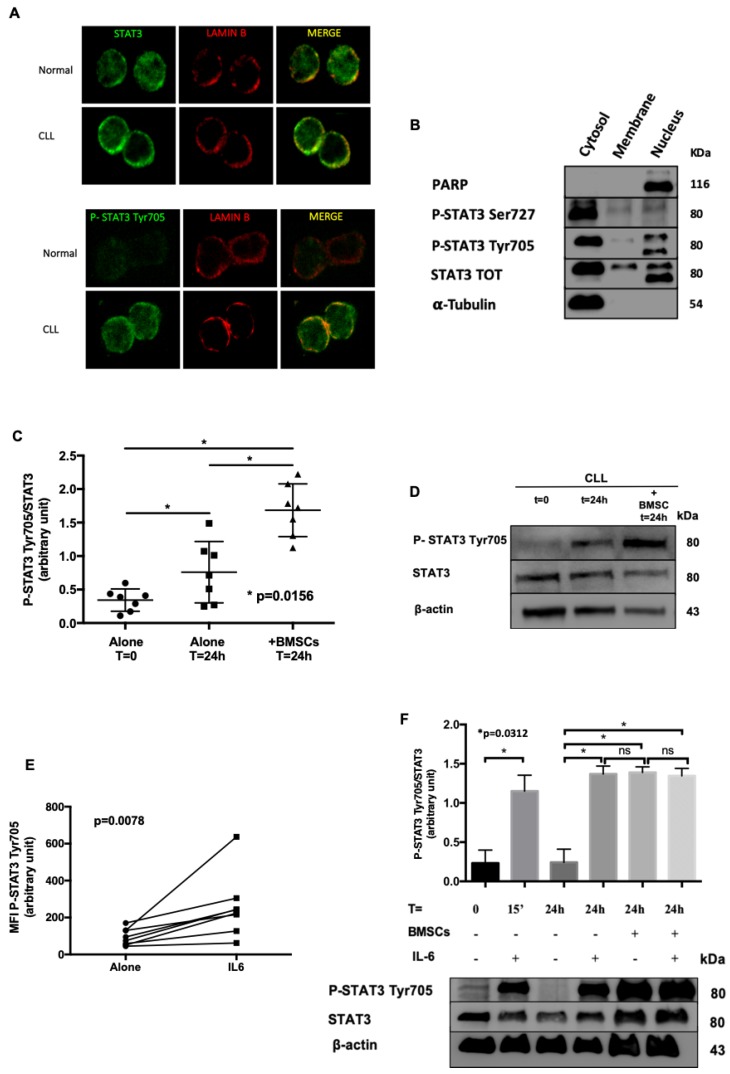
Localization and Modulation of STAT3 Tyr705 phosphorylation. (**A**) Confocal microscopy analysis of STAT3 and P-STAT3 Tyr705 (green) in normal and CLL cells obtained from peripheral blood. Lamin B (red) was used to delimit the nucleus. Both STAT3 and P-STAT3 Tyr705 are localized into the nucleus (magnification 60x). (**B**) WB after sub-cellular fractionation. In CLL P-STAT3 Tyr705 shows a relevant nuclear localization, where P-STAT3 Ser727 was found both in the nucleus and the cytosol of leukemic cells. PARP and α-tubulin were used as positive markers for nuclear and cytoplasmic compartments, respectively. (**C**,**D**) CLL were cultured for 24 h alone or on a BMSC layer. The graph reports the densitometric analysis of P-STAT3 Tyr705/STAT3 ratio from 7 cases. The phosphorylation of STAT3 on Tyr 705 increases after 24 h of culture, likely from autocrine and paracrine release of IL-6 by CLL [14], and more significantly after co-culture with BMSCs. The panel B shows a representative WB. (**E**) Flow cytometry data comparison of P-STAT3 Tyr705 between CLL cells, alone and treated with IL-6. As expected, IL-6 increased the intensity of STAT3 phosphorylation on Tyr705. (**F**) P-STAT3 Tyr7057/STAT3 WB densitometry ratio after 15 min and 24 h with (+) or without (−) BMSCs and IL-6. The panel shows a representative WB. MFI = Median Fluorescence Intensity; BMSCs = Bone marrow mesenchymal stromal cells; IL-6 = Interleukin-6.

Subsequently, we studied STAT3 sub-cellular localization by confocal microscopy. We observed that at basal condition STAT3 was phosphorylated in Tyr705, both in the nucleus as well as in the cytosol of leukemic cells, but not in normal B cells (Figure 2A). To further support our findings, we performed sub-cellular protein fractionation, detecting again STAT3 Tyr705 in both compartments of neoplastic cells (Figure 2B). Conversely, STAT3 Ser727 was located mainly in the cytoplasm of CLL cells (Figure 2B). The purity of the different sub-cellular fractions was assessed through the presence or absence of PARP (Poly ADP-Ribose polymerase) and α-Tubulin proteins, localized in nucleus and cytosol, respectively. As already known [1], the phosphorylation of STAT3 on Tyr705 regulates the activity of STAT3, its nuclear translocation, thus favoring the transcription of pro-survival and anti-apoptotic genes. 

Altogether, the overexpression of JAK2 and STAT3 in CLL cells, the phosphorylation, and the nuclear localization of P-STAT3 Tyr705 at basal condition, suggest that JAK2/STAT3 pathway is constitutively active in CLL and might play a key role in its pathogenesis and growth.

### 2.2. Bone Marrow Microenvironment Sustains STAT3 Activation

To understand whether the bone marrow environment might regulate STAT3 activation, we performed further experiments taking advantage of patients’ derived BMSCs available in our laboratory [14]. By WB, we evaluated STAT3 phosphorylation on Tyr705 in different conditions: (i) freshly isolated CLL cells; (ii) CLL cells cultured in medium alone for 24 h; (iii) CLL cells cultured for 24 h on a layer of BMSCs. As expected [14,23], BMSCs improved the survival of CLL cells. Noteworthy, we also showed that the constitutive phosphorylation of STAT3 on Tyr705 increased when CLL cells were cultured for 24 h in the presence of BMSCs (Medium alone 0.76 ± 0.17 vs. BMSCs 1.68 ± 0.15; Wilcoxon matched-pairs test, *p* = 0.0156) (Figure 2C,D). Since IL-6 is one of the main activator of JAK2/STAT3 pathway, and one of the most representative cytokines released by the bone marrow in CLL [14], we analyzed the influence of IL-6 on CLL cells. As shown in Figure 2E, by flow cytometry we demonstrated a significant increase of STAT3 phosphorylation on Tyr705 in response to IL-6 (N = 8, Wilcoxon test, *p* < 0.0078). These data were also supported by WB, showing that IL-6 up-regulates P-STAT3 Tyr705 (N = 5; Wilcoxon matched-pairs test, *p* < 0.05) at levels comparable to those obtained when CLL cells were cultured on BMSC layers (Figure 2F). 

### 2.3. Inhibition of JAK2/STAT3 Axis Induces Apoptosis of CLL Cells

**Figure 3:**
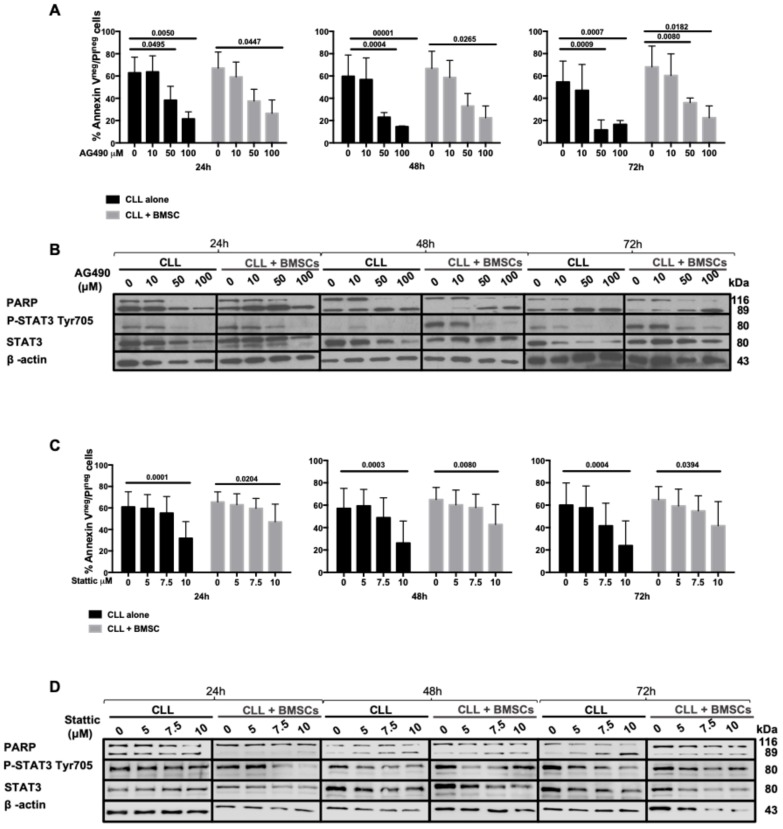
Inhibition of JAK2/STAT3 pathway by AG490 and Stattic. (**A**), (**C**) CLL cells viability after in vitro treatment for 24, 48, and 72 h with AG490 (**A**) and Stattic (**C**), as assessed by Annexin V/PI flow cytometric test in the presence (light grey histograms) or absence (black histograms) of BMSCs. (**B**), (**D**) WB analysis of PARP cleavage, STAT3 phosphorylation in Tyr705 and total STAT3 after in vitro treatment for 24, 48, and 72 h with AG490 (**B**) and Stattic (**D**) with/without BMSCs. BMSCs: bone marrow mesenchymal stromal cells.

B cells from CLL patients were cultured in medium alone or in the presence of increasing concentrations of AG490, a JAK2 inhibitor, in order to inactivate the JAK2/STAT3 axis and to assess the cell viability after 24, 48, and 72 h of treatment. We observed that AG490 was able to trigger a time- and dose-dependent apoptosis in CLL cells, as shown by Annexin V/PI tests and the cleavage of PARP by WB (Figure 3A,B), and the down-regulation of Mcl-1 and Bcl-2 proteins (Figure S4A). In vitro treatment with AG490 at 50 μM significantly decreased alive cells (Annexin V−/PI−, Figure 3A) being 39%, 22% and 18% after 24, 48, and 72 h, respectively (*p* < 0.01 at 24 h; *p* < 0.0001 at 48 h and 72 h). The same experiments were performed with increasing concentrations of Stattic, a STAT3 dimerization/nuclear translocation inhibitor [24]. As shown in Figure 3C,D, Stattic was able to decrease the viability of CLL cells in a dose-dependent manner but less effectively than AG490. To mimic the bone marrow tumor microenvironment, the above-mentioned experiments were performed in the presence of BMSCs recovered from CLL patients, showing that both AG490 and Stattic were able to induce apoptosis in CLL cells in a dose-dependent manner even in the presence of bone marrow microenvironment protection (Figure 3). 

Interestingly, AG490 treatment was significantly more toxic on CLL cells, impairing their vitality, with respect to normal B lymphocyte (Figure S4B). High doses of AG490 decrease the viability of B lymphocytes by a fold rate of 20% as compared to almost 40% in CLL cells (*p* < 0.01, Mann–Whitney test).

### 2.4. AG490 Treatment Activates SHP-1 Phosphatase and Inhibits Lyn Kinase in CLL Cells

**Figure 4:**
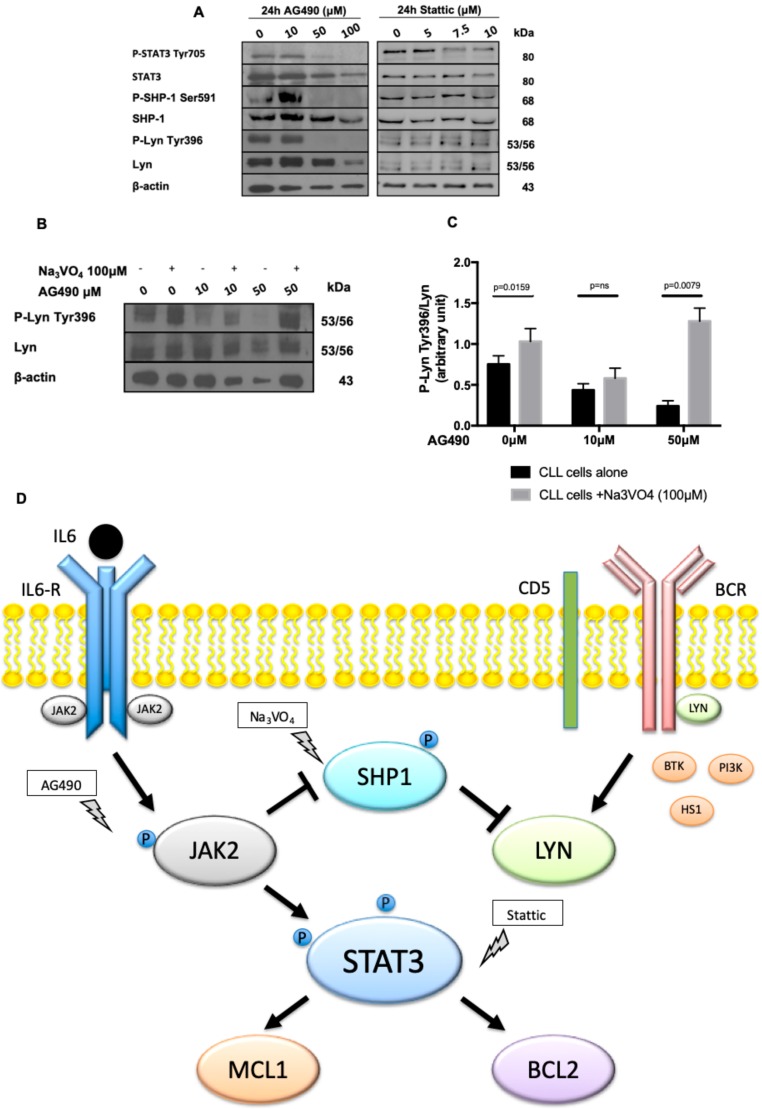
JAK2/STAT3/Lyn cross-talk assessment in CLL cells. (**A**) A representative blot of CLL cells cultured for 24 h with increasing doses of AG490 or Stattic. In vitro treatment with AG490, but not Stattic, caused the down-regulation of P-STAT3 Tyr705, P-SHP-1 Ser591, and P-Lyn Tyr396. (**B**) CLL cells were cultured with (+)/without (-) 100 μM of Na_3_VO_4_, a known SHP-1 inhibitor [27], and increasing concentrations of AG490 for 24h. Immunostaining with anti-Lyn Tyr396, anti-Lyn and anti-β-actin antibodies highlights Na_3_VO_4_ capability to restore Lyn phosphorylation on Tyr396, previously down-regulated by AG490 administration. (**C**) Histograms median P-Lyn Tyr396/Lyn ratio of WB analysis densitometry from 5 cases. Black bars: CLL cells alone. Grey bars: CLL cells + Na_3_VO_4_. Na_3_VO_4_: sodium orthovanadate. (**D**) A schematic representation of the molecules involved in JAK2/STAT3/Lyn cross-talk in CLL, after AG490, Stattic, and Na_3_VO_4_ treatment.

We previously demonstrated that in CLL lymphocytes the Src kinase Lyn is constitutively active, phosphorylated in Tyr396 and its inhibition triggers CLL cells apoptosis [8,25]. Moreover, our group demonstrated that in CLL, Lyn activation/phosphorylation on Tyr396 is maintained by the phosphatase SHP-1 constitutively phosphorylated (i.e., inhibited) on Ser591 [18,19]. To better understand the mechanism of CLL cell apoptosis induced by AG490 and Stattic, we co-cultured neoplastic B cells from 9 CLL patients with increasing concentrations of AG490 and Stattic, and by WB we assessed the expression and phosphorylation status of SHP-1 and Lyn. Starting from 50μM in vitro treatment with AG490 was able to de-phosphorylate STAT3 on Tyr 705 (Figure 4A) and to mediate the dephosphorylation of both SHP-1 on Ser591, thus activating the phosphatase, and Lyn on Tyr396 (Figure 4A). On the contrary, the treatment with Stattic did not affect Lyn and SHP-1 phosphorylation (Figure 4A), likely because it acts inhibiting STAT3 dimerization and/or its nuclear translocation [24] downstream to JAK2 (Figure 4D). To further confirm the link between JAK2 inhibition by AG490, SHP-1 activation and Lyn dephosphorylation/deactivation, we co-treated CLL cells with AG490 and sodium orthovanadate (Na_3_VO_4_, 100 μM), a known inhibitor of SHP-1 [26,27], in order to restore Lyn activation (resulting from the inactivation of SHP-1 phosphatase). As shown in Figure 4B, Na_3_VO_4_ was able to restore the phosphorylation of Lyn on Tyr396 phosphorylation impaired by AG490 treatment (median densitometry P-Lyn-Tyr396/Lyn was 0.2589 after treatment with AG490 50 μM vs. 1.308 with AG490 50 μM + Na_3_VO_4_, *p* = 0.0079, Figure 4C), supporting the hypothesized mechanism of regulation between JAK2-SHP1 and Lyn.

### 2.5. JAK2/STAT3 Inhibitors Enhance Ibrutinib Activity within the Bone Marrow Microenvironment

Even if ibrutinib is broadly active in CLL patients, the rate of complete remission with this drug is low, mainly due to the persistence of resistant clones within the bone marrow. We hypothesized that BMSCs, through the activation of JAK2/STAT3 pathway, may be involved in the modulation of ibrutinib activity. To address this point, we treated in vitro CLL cells for 24 h with either ibrutinib, JAK2/STAT3 inhibitors (i.e., AG490 or Stattic), or both drugs, in the presence or not of BMSC layers. 

**Figure 5:**
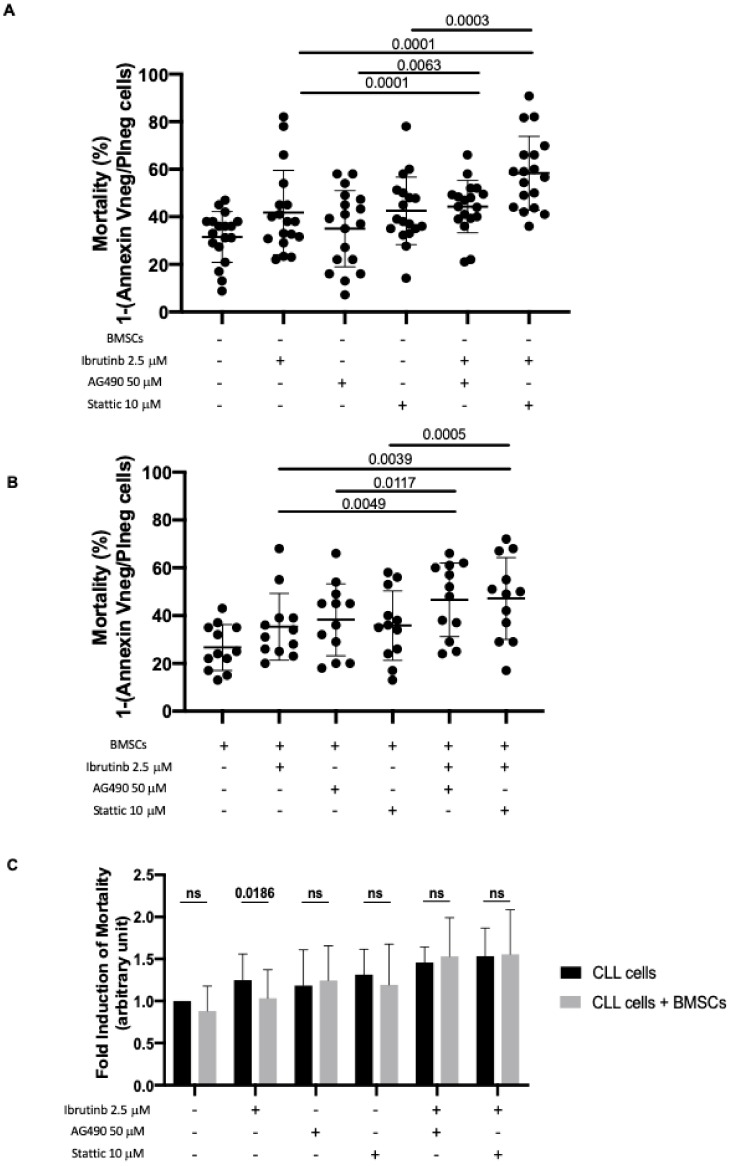
Combined effect of Ibrutinib with JAK2/STAT3 inhibitors. (**A**) Mortality of leukemic cells cultured in medium with ibrutinib, AG490, and Stattic, as single agent and in combination of ibrutinib + AG490 or ibrutinib+Stattic. N = 18. Co-treatment of ibrutinib+AG490 or ibrutinib + Stattic increased the mortality compared to ibrutinib alone. Mortality was calculated as 1–Annexin Vneg/PIneg cells. (**B**) Mortality of leukemic cells cultured in the presence of BMSCs with ibrutinib, AG490, and Stattic, as single agent and combination of ibrutinib+AG490 or ibrutinib+Stattic. N = 12. Co-treatment of ibrutinib + AG490 or ibrutinib+Stattic increased the mortality compared to ibrutinib used as single agent. Mortality was calculated as 1–Annexin Vneg/PIneg cells. (**C**) Fold induction of CLL cell mortality, calculated as ratio with untreated cells. Mortality was evaluated by flow cytometry and Annexin V/PI test, and calculated as 1–Annexin Vneg/PIneg cells. N = 12 Black bars: CLL cells in medium alone. Gray bars: CLL cells in the presence of BMSCs. The mortality caused by ibrutinib is hampered by BMSC co-cultures, and co-inhibition of JAK2/STAT3 pathways overcomes bone marrow support. BMSCs: Bone marrow mesenchymal stromal cells.

The mortality rate of CLL leukemic cells (mortality was calculated as 1–Annexin Vneg/PIneg cells) cultured in medium alone and incubated in the presence of ibrutinib only increased significantly when AG490 or Stattic were added to the culture. The data are reported as percentage of cell death and as fold induction between cells cultured in the presence of compound with respect to cells cultured alone (Figure 5A) with and without BMSCs (Figure 5B). Specifically, the mortality rate of CLL leukemic cultured in medium alone+ibrutinib was 34% with respect to cells cultured in medium alone, that was 28% (*p* = 0.0001; fold induction 1.32); this value increased in the presence of AG490 to 46% (*p* = 0.0001, fold induction of 1.81) and in the presence of Stattic to 55% (*p* = 0.0001; fold induction of 2.00; Figure 5A). Furthermore, the co-culture of CLL cells with BMSCs (Figure 5B) showed a slight effect of both AG490 and Stattic on leukemic cell viability. In particular, the viability ranged from 35% (*p* = 0.0015; fold induction of 1.35) in cells cultured in the presence of BMSCs and ibrutinib to 47% (*p* = 0.0049; fold induction of 1.79) following the addition of AG490 in cell culture and to 47% (*p* = 0.0039; fold induction of 1.80) following the addition of Stattic.

When CLL cells were cultured on BMSC layers, the mortality caused by ibrutinib is hampered (Figure 5C, column ibrutinib 2.5 μM, fold induction, alone 1.25 ± 0.31 vs. BMSCs 1.03 ± 0.34, *p* = 0.0186, Wilcoxon matched-pairs signed rank test), likely related to activation of JAK2/STAT3 signaling as previously demonstrated in this work (Figure 2). However, the effect was abrogated when cells were treated with the drug combinations, ibrutinib+AG490 or ibrutinib+Stattic, even in the presence of bone marrow BMSC layers (Figure 5C columns ibrutinib+AG490 and ibrutinib+Stattic). In fact, the mortality of CLL cells treated with both inhibitors increased by a fold rate of almost 50% and was not rescued by BMSCs, as opposite to cells treated with ibrutinib alone (Figure 5C). These data suggest the ability of JAK2/STAT3 inhibitors to overcome CLL bone marrow niche protection from ibrutinib killing.

## 3. Discussion

We herein demonstrated the involvement of JAK2/STAT3 pathway in CLL cell survival and its modulation by bone marrow stromal cells. We showed that STAT3 is constitutively active in CLL cells, being phosphorylated on Ser727 and Tyr705, and that the block of JAK2/STAT3 pathway by specific inhibitors affects the viability of the neoplastic clone, overcoming the bone marrow environmental protection. Finally, the ability of AG490 and Stattic to strengthen the effect of ibrutinib offers a starting point for the development of new therapeutic strategies in CLL.

The JAK2/STAT3 axis is one of the most recognized signaling pathway, whose constitutive activation occurs with high frequency in several hematopoietic diseases and solid tumors [28,29,30,31]. STAT3 is a key transcriptional factor in CLL, being involved in the regulation of anti-apoptotic proteins [15], microRNA [32], metabolism [33], and expression of immune checkpoint molecules [34]. Hazan-Haley et al. [35] first demonstrated that in CLL STAT3 is constitutively phosphorylated on Ser727, regardless of clinical parameters, being translocated into the nucleus by the karyopherin-beta nucleocytoplasmic system and bound to p21 and c-Myc genes. Serine phosphorylation of STAT3 is regulated by an aberrant cytoplasmic complex of CD5, BLNK, and CK2 (a pleiotropic serine/threonine kinase [36]) [37]. Moreover, it has been published that both IL-6, through the recruitment of JAK2, and the BCR, through a JAK2-independent mechanism, can promote STAT3 phosphorylation on Tyr705 [38]. Whereas IL-6 induced STAT3-Tyr705 phosphorylation within a few minutes, the stimulation of the BCR with anti-immunoglobulin M (IgM) antibodies required a few hours, since it involves the transcriptional activity of NF-κB. Remarkably, prolonged incubation with anti-IgM antibodies induces transcription and secretion of IL-6, further promoting the phosphorylation of STAT3 on tyrosine residues. This effect is counteracted by the JAK1/2 inhibitor ruxolitinib [38].

We demonstrated that STAT3, as well as JAK2, are over-expressed in CLL cells with respect to normal B lymphocytes, regardless of clinical and biological prognostic markers. We confirmed the well described phosphorylation on Ser727 [8,26] (Figure S1C), but we also identified a constitutive phosphorylation of STAT3 on Tyr705 in patients with progressive disease (Figure 1E,F–I). Since the levels of IL-6, one of the main activator of JAK2/STAT3 pathway, are significantly increased in serum of CLL patients and correlate with adverse clinical features and shorter survival [39], it is not surprising that we found higher P-STAT3-Tyr705 in patients with progressive disease requiring treatment. Moreover, a significant amount of phosphorylated STAT3 on Tyr705 was localized in the nucleus of CLL cells (Figure 1H,I), where this protein can exert its function as a transcription factor through the activation of pro-survival genes [40,41] and anti-apoptotic factors [3,4], such as Mcl-1 and Bcl-2 [7], both over-expressed in CLL (Figure S4), underpinning the role of STAT3 in CLL pathogenesis. We also showed that in vitro treatment with the JAK2 inhibitor AG490, or the STAT3 inhibitor Stattic was able to trigger time- and dose-dependent apoptosis of CLL cells. 

We showed that in vitro treatment with AG490, but not with Stattic, caused the dephosphorylation of STAT3 on Tyr705 (Figure 3B–D), the down-regulation of Mcl-1 and Bcl-2 (Figure S3), but also of Lyn on Tyr396 (Figure 4A). Lyn is the most important Src kinase in CLL, aberrantly localized in the cytosol, part of another cytosolic complex along with HS1-SHIP1-HSP90, constitutively phosphorylated on Tyr396, its inhibition/dephosphorylation triggering apoptosis [8,25]. This basal phosphorylation of Lyn is maintained by the inactivation of the tyrosine phosphate SHP-1, which is phosphorylated/inhibited on Ser591 [27]. The activation of the phosphatase PPA2 mediates the dephosphorylation/activation of SHP-1 and, consequently, Lyn dephosphorylation/inactivation on Tyr396 and CLL cell death [27,42]. According to data from the literature, we observed that JAK2 inhibition by AG490 triggered the dephosphorylation of SHP-1 on Ser591 (Figure 4A), likely through PP2A activation [43], of Lyn on Tyr396, and CLL apoptosis. This scenario (Figure 4D) has been experimentally corroborated by incubating CLL cells with AG490 together with a SHP-1 inhibitor, i.e., Na_3_VO_4_, which rescues Lyn phosphorylation on Tyr396 (Figure 4B,C). Consistent with the literature, the basal STAT3 phosphorylation on Tyr705 increased when CLL cells were co-cultured on BMSC layers or incubated with IL-6. In addition, Levidou et al. recently reported that STAT3 is constitutively phosphorylated at Tyr705 in CLL cells resident in lymph nodes [44].

The interactions between CLL cells and the microenvironment are crucial for both the survival and the progression of the malignant clones. Co-cultures of CLL cells with BMSC, the most abundant stromal cells in the bone marrow, protect CLL cells from spontaneous death through cell-cell contact and the release of cytokines such as IL-6 [14,23]. Our group and others has previously demonstrated that BMSCs are not only able to protect CLL cells from spontaneous apoptosis, but they also rescue leukemic cells after treatment, in vivo or in vitro, for example with traditional drugs such as Fludarabine and Cyclophosphamide [14,23,45]. Hence the need to find new drugs able to counteract BMSCs effects.

In CLL, when ibrutinib is used as single agent, the rate of complete remission is low or is achievable after several months [18], mainly due to the persistence of leukemic cells within the bone marrow. On the basis of the up-regulation of STAT3 pathways in CLL by BMSCs, we hypothesized that JAK2/STAT3 pathway might hamper ibrutinib activity in the bone marrow. We demonstrated that BMSCs were able to impair ibrutinib-induced in vitro CLL mortality (Figure 5), and the combination of JAK2/STAT3 inhibitors, either AG490 or Stattic, plus ibrutinib was able to counteract the pro-survival effect mediated by BMSCs and increase CLL mortality by almost 50% (Figure 5) probably inducing apoptosis in that portion of cells that ibrutinib had not been able to kill because, de facto, protected by microenvironment. Other authors have successfully managed to overcome the protection of BMSCs by targeting PI3Kγ, PKCβ or FAK [45,46]. Given the low fraction of complete remission after ibrutinib mainly due to the persistence of cells in the bone marrow and the ability of JAK2 and STAT3 inhibitors to overcome the protection of the bone marrow microenvironment, this work opens interesting perspectives to study therapeutic strategies aimed at eradicating the leukemic clone with the ultimate goal of patient healing.

Interestingly, two phase II clinical trials explored ruxolitinib, a JAK1-2 inhibitor approved for the treatment of primary myelofibrosis, in CLL. While the first trial reported a decrease of lymph node size in almost 50% of patients, the study was stopped early due to a high incidence of severe anemia [47]. The second trial showed that ruxolitinib significantly improved disease-related symptoms, decreased serum cytokines, and caused leukemic cell mobilization from lymphoid organs [48]. These trials provide preliminary observation of the positive effect of JAK2/STAT3 targeting in CLL, either alone or in combination with BTK inhibitors.

## 4. Methods

### 4.1. Ethics Statement

This study was authorized by the Padua Ethics Committee (Approve Code:n. 3529/AO/14) and written informed consent has been provided to all CLL patients included in the study, according to the Declaration of Helsinki.

### 4.2. Patients, Cell Separation, and Culture Conditions

Peripheral blood B lymphocytes were derived from 23 age-matched healthy donors and 66 patients diagnosed with treatment-naïve CLL followed at Padua University Hospital and diagnosed according to international workshop of CLL 2018 guidelines [49]. The biological and clinical characteristics of CLL patients were evaluated as previously reported [50,51] and listed in Table 1.

The purity of the obtained peripheral blood cells was at least 95% (CD19+), as assessed by flow cytometry. Purified cells (2 × 10^6^ cells/mL) were cultured in suspension in RPMI-1640 medium (EuroClone; Milan, Italy) supplemented with 10% heated inactivated Fetal Calf Serum (FCS; Invitrogen, Paisley, UK), 2 mmol/L-glutamine, 100 μg/ml penicillin and 100 μg/mL streptomycin in 24-well plates, at 37 °C in a humidified atmosphere containing 5% CO_2_. In different experiments, cells were treated with AG490 (Selleck Chemicals; Houston, TX, USA) for 24, 48, and 72 h at 10, 30, 50, and 100 μM; Stattic (Selleck Chemicals) at 5, 7.5, and 10 μM for 24, 48, and 72 h. In some experiments, sodium orthovanadate (Na_3_VO_4_) 100 μM and IL-6 25 ng/mL (Sigma-Aldrich; St. Louis, MO, USA) were used. Co-cultures of CLL cells and BMSCs, ratio 20:1, were done in 12 well plates with RPMI 1640, antibiotics, and 10% FBS. BMSCs from CLL cells were previously obtained as described by Trimarco et al. [14].

### 4.3. Cell Viability Testing

After incubation with AG490 and Stattic, apoptosis was assessed using the Annexin V-FITC kit (Immunostep; Salamanca, Spain), as previously described [14].

### 4.4. Western Blotting Analysis

Cells (5 × 10^5^ for each assay) were prepared by cell lysis, subjected to sodium dodecyl sulphate polyacrylamide gel electrophoresis (SDS/PAGE), transferred to nitrocellulose membranes, as previously described [52], and immunostained with antibodies to p-STAT3 Tyr705, p-STAT3 Ser727, STAT3, PARP, JAK2 (Cell Signaling Technology Inc.; Danvers, MA, USA); p-SHP-1 Ser591, SHP-1 (Millipore; Billerica, MA, USA); p-Lyn Tyr396 (Epitomics Onc.; Burlingame, CA, USA); Lyn (Santa Cruz Biotechnology; Dallas, TX, USA); α-Tubulin and β-Actin (Sigma-Aldrich). Original WBs are displayed in the Supplementary Materials (Figure S5).

### 4.5. Flow Cytometry Phospho-Protein Analysis

Cells (2 × 10^6^ for each assay) were collected after 15 min and fixed in Lyse/Fix Solution (Becton Dickinson, BD; Franklin Lakes, NJ, USA) for 10 min at 37 °C, washed and permeabilized with a Perm Buffer (0.5% saponin, 5% FCS, 10mM N-2-hydroxyethylpiperazine-N’-2-ethanesulfonic acid) for 20 min at room temperature. Samples were washed and incubated with the antibody of interest (anti-P-STAT3 Tyr705 PE or anti-P-STAT3 Ser727 FITC) for 30 min at 4 °C and analyzed with Fluorescence-Activated Cell Sorting (FACS) Canto II (BD) [53]. To evaluate the phosphorylated protein amount, the difference between the MFI of the sample labelled and the MFI of the Fluorescence *Minus* One (FMO) control was performed.

### 4.6. Confocal Microscopy Analysis

Cells were collected and then stained, as previously described [25], with antibodies against Lamin B (Santa Cruz), STAT3 and P-STAT3 Tyr705 (Cell Signaling) diluted 1:250. The secondary antibodies used were anti-mouse-FITC (STAT3 and P-STAT3-Tyr705) and anti-goat-Alexa 594 (Lamin B). Fluorescence was detected using the UltraView LCI confocal system (Perkin Elmer; Walthaw, MA, USA).

### 4.7. Sub-Cellular Fractionation

The sub-cellular fractions were obtained using a commercial kit (Thermo Scientific, Rockford, IL, USA) using detergents which could separate cytoplasmic, membrane and nuclear proteins [54]. 10^7^ lymphocytes were centrifuged and incubated with different buffers following manufacture’s protocol. 

### 4.8. Statistical Analysis

GraphPad Prism v. 7 (GraphPad Software, La Jolla, CA, USA) was used to perform the data analyses. Data are reported as mean ± standard deviation (SD) and were considered statistically significant when p values were <0.05. Data were evaluated for their statistical significance with appropriate tests: Mann–Whitney test was used to compare continuous variables; differences between groups were tested by applying the Analysis of Variance (ANOVA or Kruskal–Wallis test) or Fisher’s exact test.

## 5. Conclusions

JAK2/STAT3 pathway was found to be active in CLL cells, and STAT3 is constitutively phosphorylated also on Tyr705. The targeting of JAK2/STAT3 pathway triggers cell apoptosis, bypassing the pro-survival stimuli provided by the bone marrow microenvironment. Furthermore, the use of ibrutinib together with JAK2 and STAT3 inhibitors increased cell mortality induced by ibrutinib alone even in the presence of BMSCs.

## Figures and Tables

**Table 1:** Biological and Clinical characteristics of the patients.

Patients	66
Median age, years (range)	72 (49–90)
Male/Female	41/25
WBCs count, ×10^9^/l (range)	49.8 (4.7–300)
Lymphocytes, % (range)	74 (46–97)
Rai Stage III–IV	13
Lymphadenopathy	21
U-IGHV*	27
TP53 deletion/mutation	8

* Immunoglobulin heavy chain variable region (IGHV) mutational status. Sequences homology <98.00%, from the corresponding germline gene, were considered mutated (M-IGHV), as opposite to unmutated (U-IGHV) cases. Cytopenia was defined as hemoglobin <100 g/L, absolute neutrophil count <1000/µL, platelets <100,000/µL.

## Supplementary Materials

The following are available online at https://www.mdpi.com/2072-6694/11/12/1939/s1, Figure S1: JAK2 and STAT3 mRNA level evaluation, Figure S2: Correlation between JAK2 and STAT3 protein expression and prognostic indexes, Figure S3: Western Blotting, flow cytometry analysis and correlation, Figure S4: AG490 effects on MCL1 and BCL2 proteins and on normal cells, Figure S5: Whole blots relative to the Western Blotting analyses, Table S1: Biological and Clinical characteristics of the patients.
