# Supplementary material for: In Chronic Lymphocytic Leukemia the JAK2/STAT3 Pathway Is Constitutively Activated and Its Inhibition Leads to CLL Cell Death Unaffected by the Protective Bone Marrow Microenvironment
Source: Cancers (Basel). 2019 Dec 4;11(12):1939. doi: 10.3390/cancers11121939 (PMC6966457; doi:10.3390/cancers11121939)
Supplement: Supplementary file 1 [file cancers-11-01939-s001.pdf]

Supplementary material

In Chronic Lymphocytic Leukemia the JAK2/STAT3 Pathway Is Constitutively Activated and Its Inhibition Leads to CLL Cell Death Unaffected by the Protective Bone Marrow Microenvironment

Filippo Severin, Federica Frezzato, Andrea Visentin, Veronica Martini, Valentina Trimarco, Samuela Carraro, Elena Tibaldi, Anna Maria Brunati, Francesco Piazza, Gianpietro Semenzato, Monica Facco and Livio Trentin

Figure S1

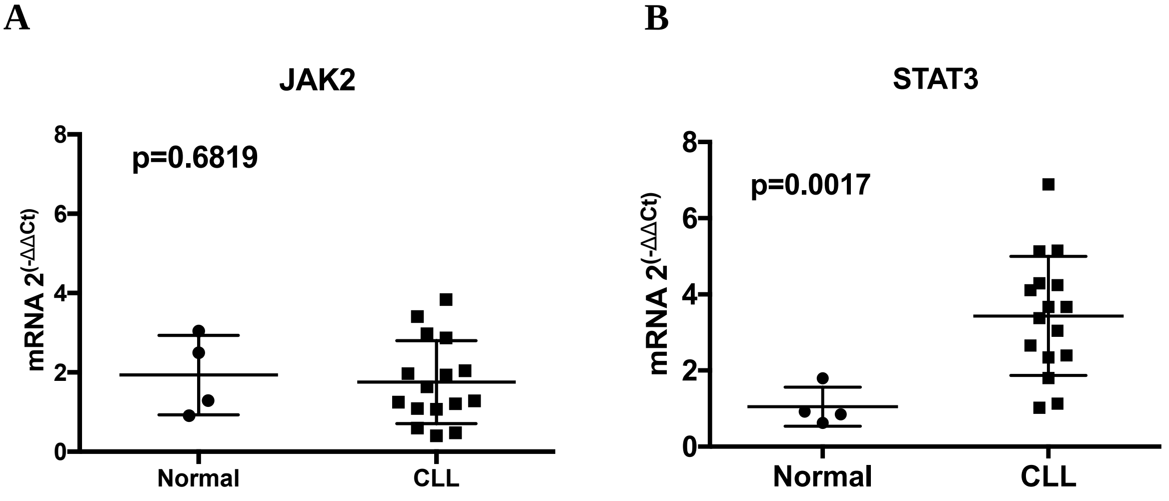

Figure S1. JAK2 and STAT3 mRNA level evaluation. We analyzed Jak2 and Stat3 mRNA levels in 4 age matched healthy subjects and 16 CLL patients. We found similar Jak2 (**A**, p=0.6819, Mann-Whitney test) but higher Stat3 (**B**, p=0.0017, Mann-Whitney test) mRNA levels in CLL cells as compared to normal B lymphocytes.

Figure S2

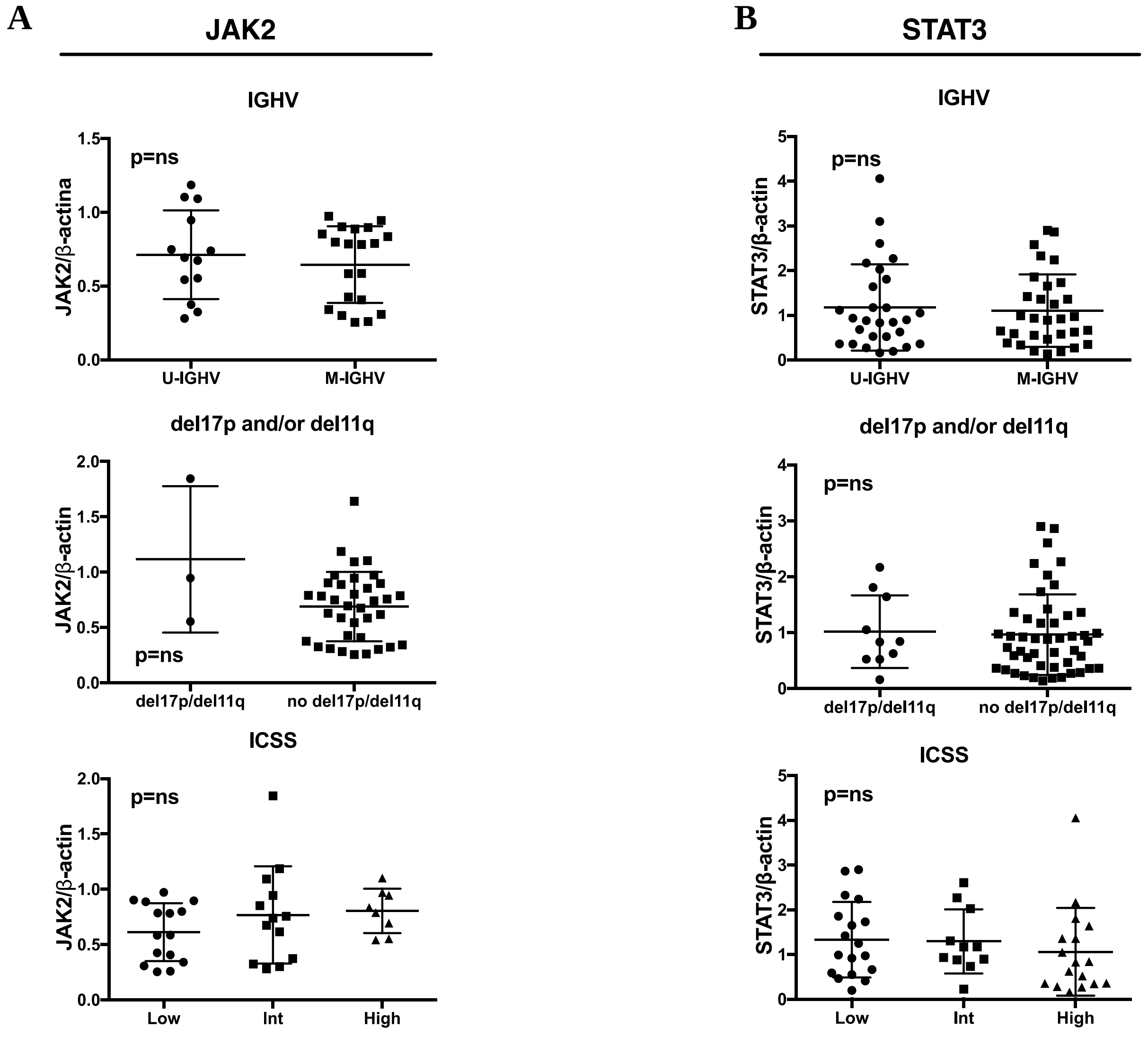

Figure S2. Correlation between JAK2 and STAT3 protein expression and prognostic indexes. A-B. Expression of JAK2 **(A)** and STAT3 **(B)** in relation to different prognostic factors such as the presence or absence of deletion of 17p13 and 11q22-23 [27], the IGHV mutational status (mutated or unmutated) [28] and the classification according to the ICSS score [29]. JAK2 and STAT3 were equally expressed in patients with favourable and unfavourable prognosis (p=ns). ICSS: CLL Integrated Scoring System; IGHV: Immunoglobulin heavy chain variable region.

Figure S3

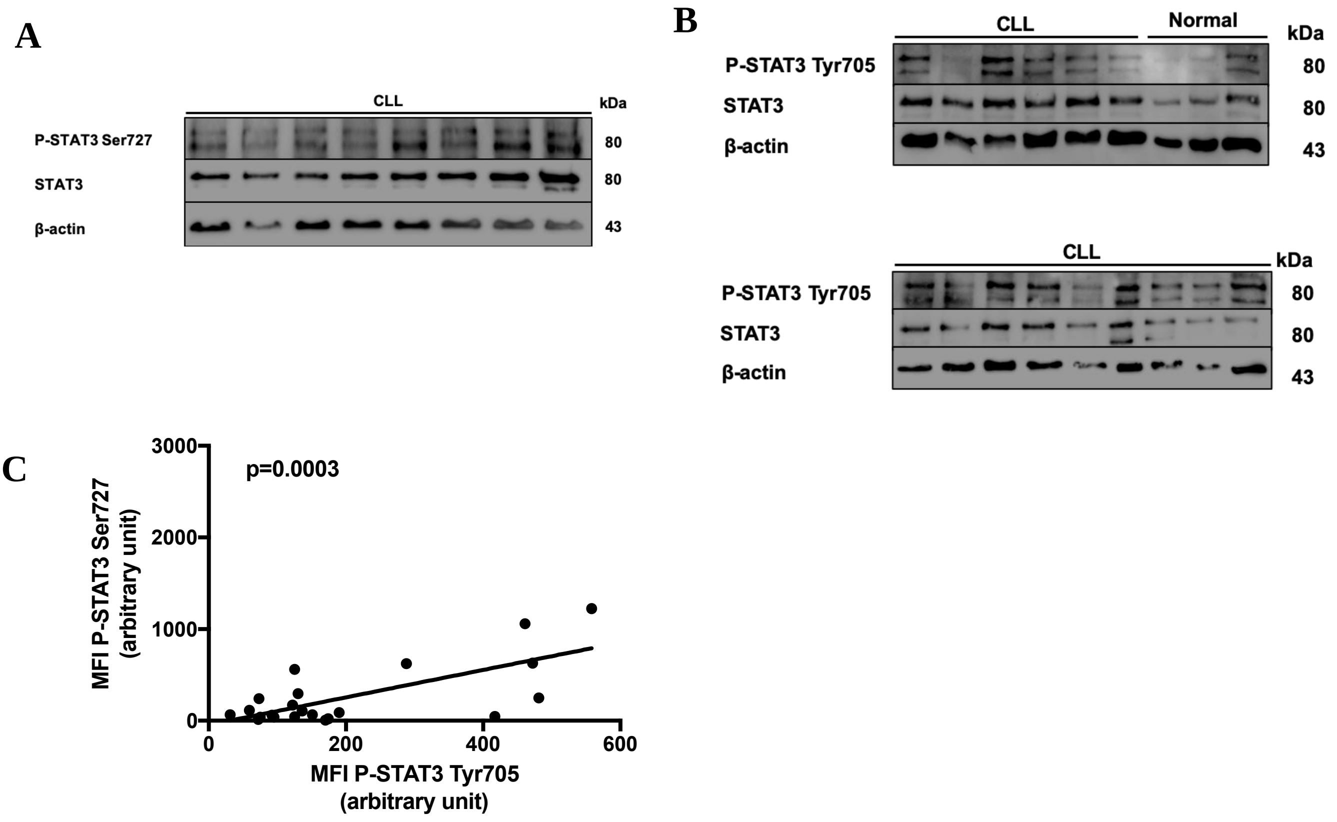

Figure S3. Western blotting, flow cytometry analysis and correlation. **A.** A representative blot from 8 CLL patients to evaluate the P-STAT3-Ser727 and STAT3. According to data coming from the literature, STAT3 was phosphorylated in Ser727 in all CLL patients at basal condition. **B.** Two representative blots to highlight STAT3 phosphorylation on Tyr705 in CLL samples and 3 age-matched health controls. At basal condition STAT3 was phosphorylated in Tyr705 in 11 out of 16 CLL cases, with different intensity. **C.** Figure shows the correlation between the MFI of P-STAT3 Tyr705 and P-STAT3 Ser727 in 23 CLL patients (Pearson correlation, p=0.0003). MFI = mean fluoresce intensity.

Figure S4

A **B**

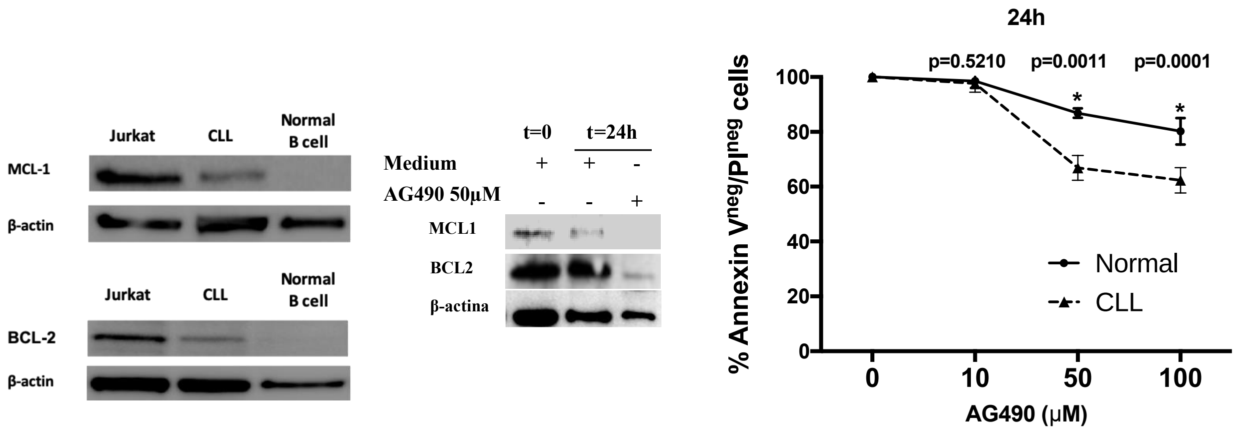

Figure S4. A. AG490 effects on MCL1 and BCL2 proteins and on normal cells. Representative blots derived from 5 cases of MCL-1 and BCL-2 protein expression in CLL, normal B cells and Jurkat cell lines (positive control). According to data from the literature BCL2 and MCL1 were found overexpressed in CLL [7]. *In vitro* treatment with AG490 for 24h led to the downregulation of both MCL-1 and BCL-2 proteins. **B.** AG490 activity on normal and CLL cells at 24h, by Annexin V/PI flow cytometry evaluation. AG490 treatment had a significant higher apoptotic effect on CLL cells with respect to normal B cells.

Figure S5. Whole blots relative to the Western Blotting analyses.

Figure 1B

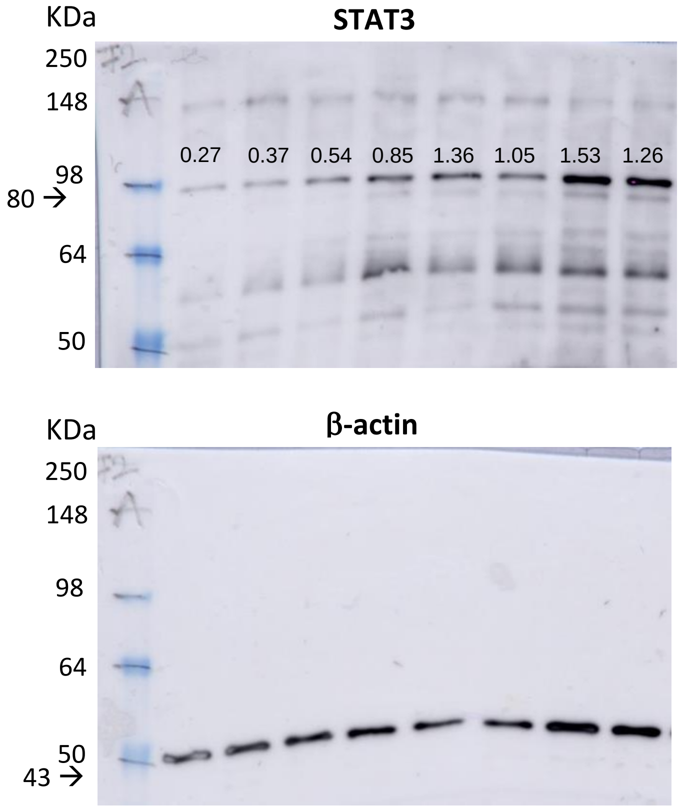

Figure 1D

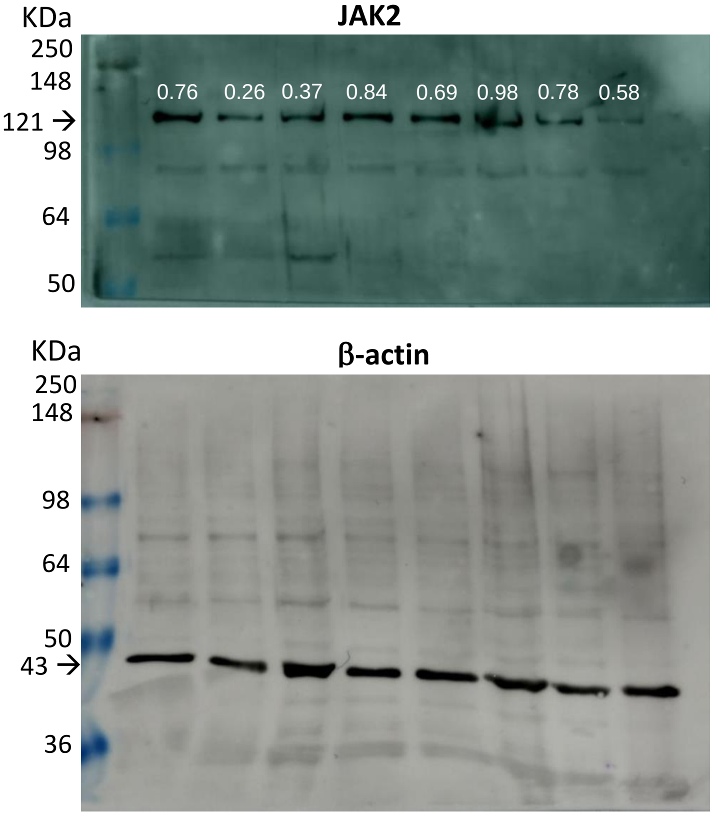

Figure 1G

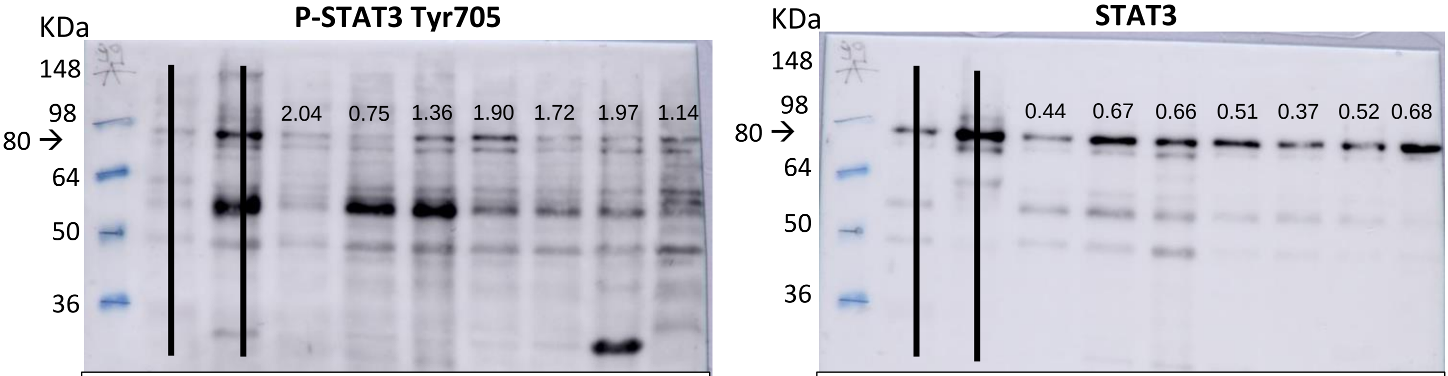

First two lines are other cellular types and they are not reported in main text.

First two lines are other cellular types and they are not reported in main text.

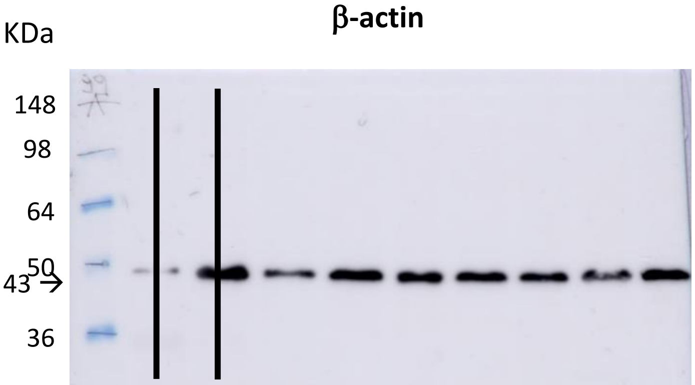

First two lines are other cellular types and they are not reported in main text.

Figure 2B

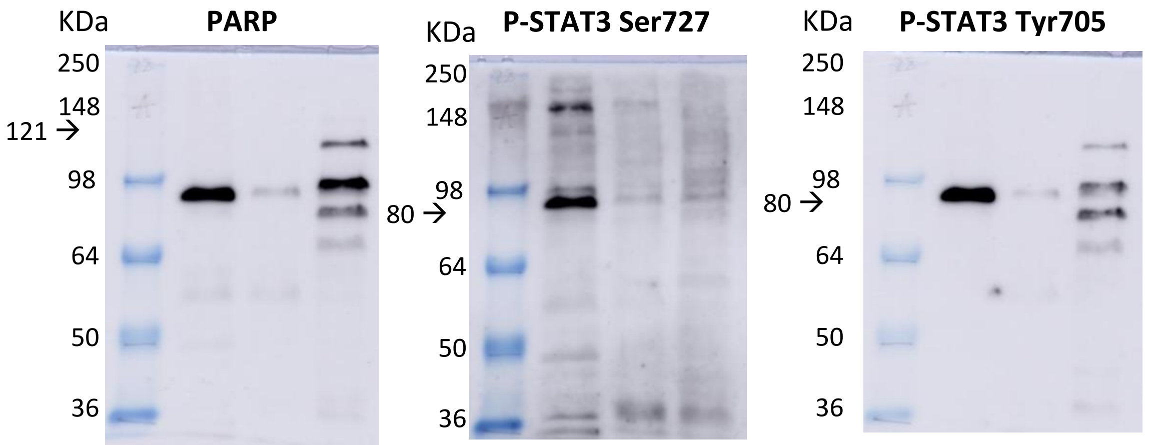

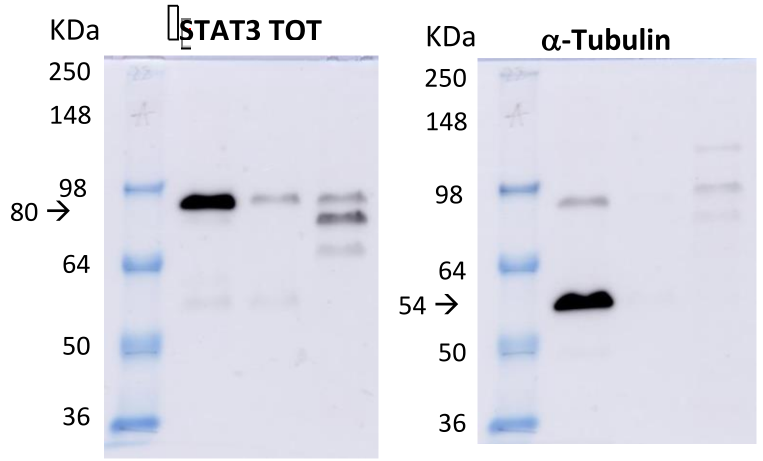

Between anti-P-STAT Tyr705 and anti-P-STAT Ser727 membrane was stripped.

Figure 2D

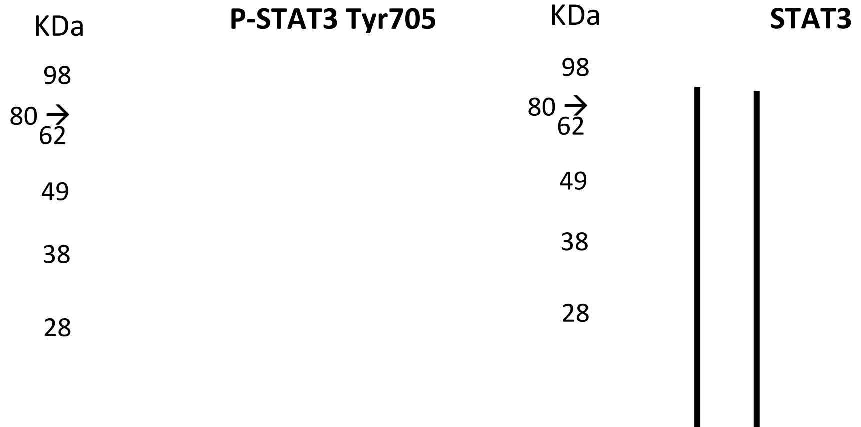

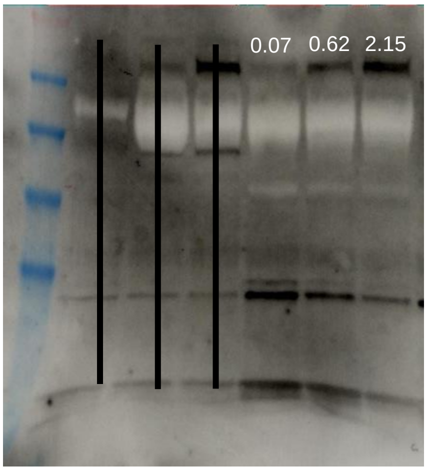

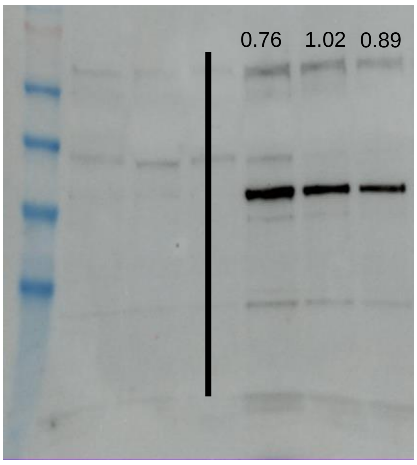

KDa

β-actin

98
62
49
43 →
38
28

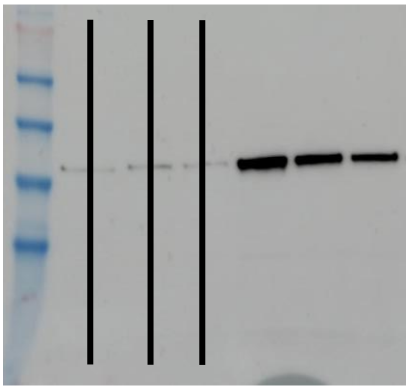

Figure 2F

KDa

P-STAT3 Tyr705

250
148
98
80 →
64
50
36

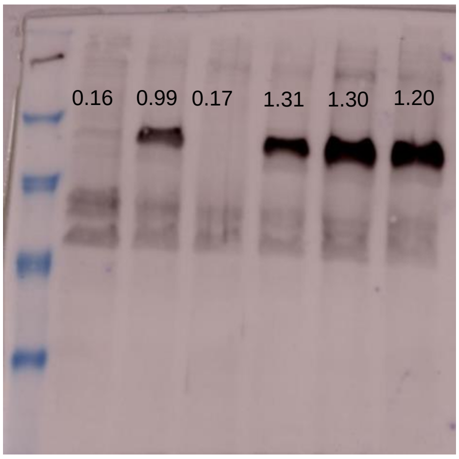

KDa

STAT3

250
148
98
80 →
64
50
36

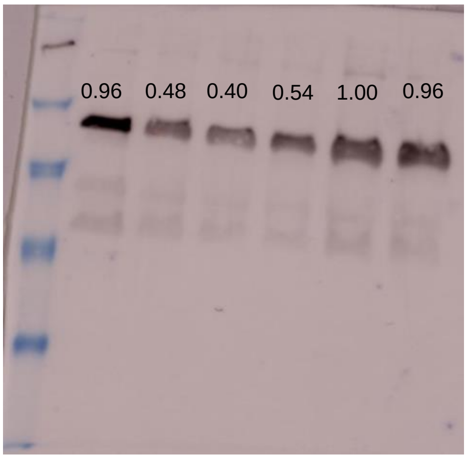

KDa

β-actin

250
148
98
64
50

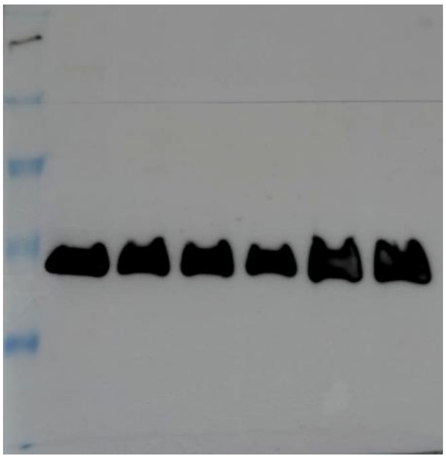

Figure 3B

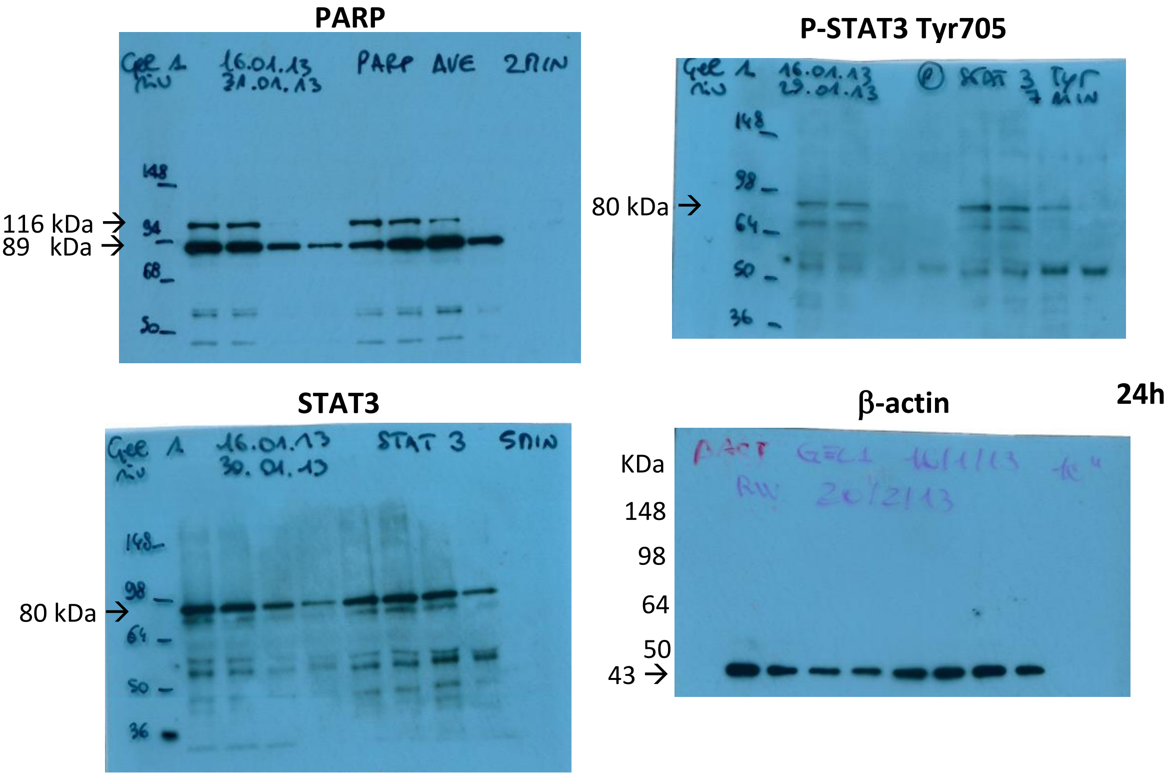
PARP
116 kDa
89 kDa
P-STAT3 Tyr705
80 kDa
STAT3
80 kDa
β-actin
24h
KDa
148
98
64
50
43

PARP

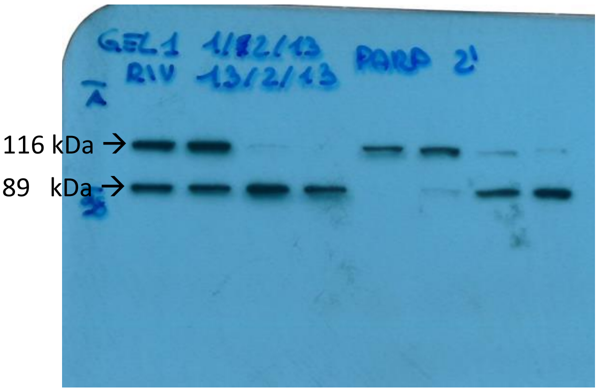

P-STAT3 Tyr705

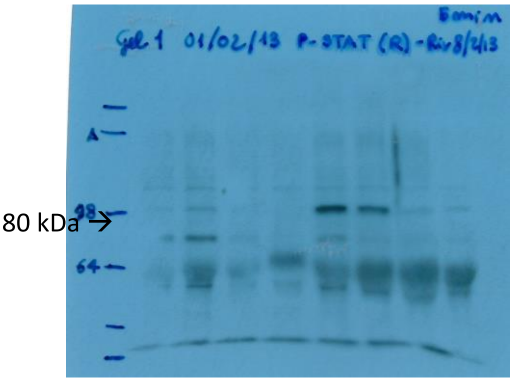

STAT3

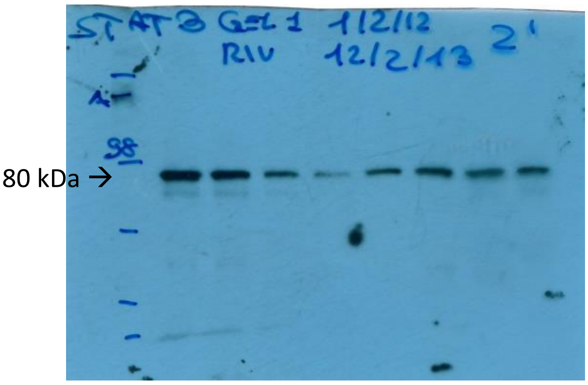

β-actin

48h

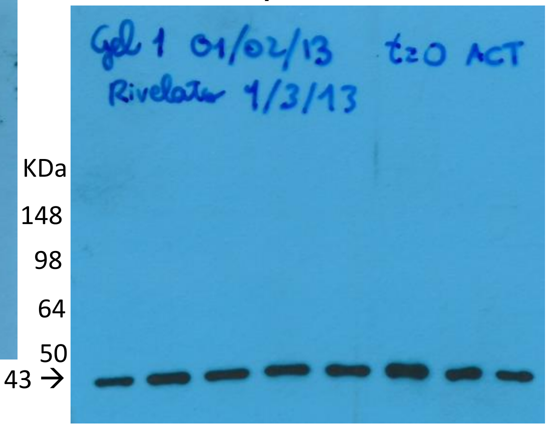

PARP

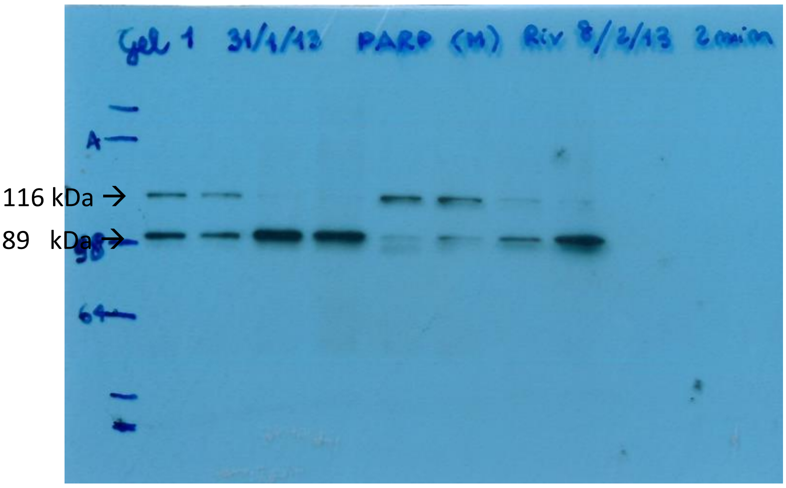

P-STAT3 Tyr705

72h

80 kDa →

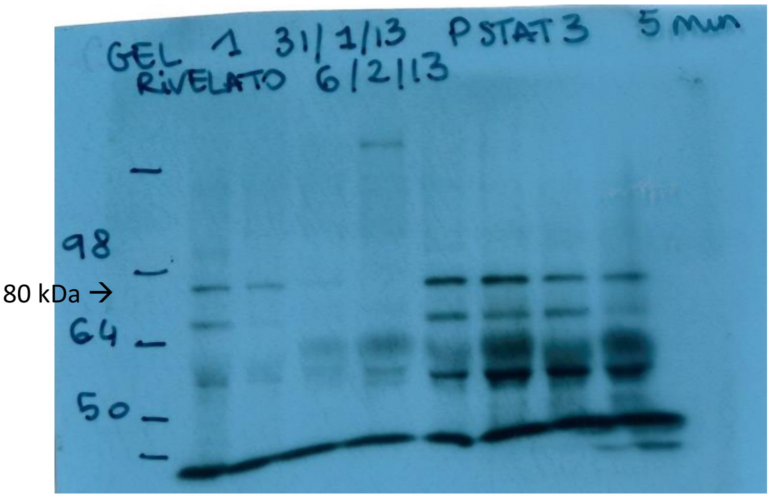

STAT3

80 kDa →

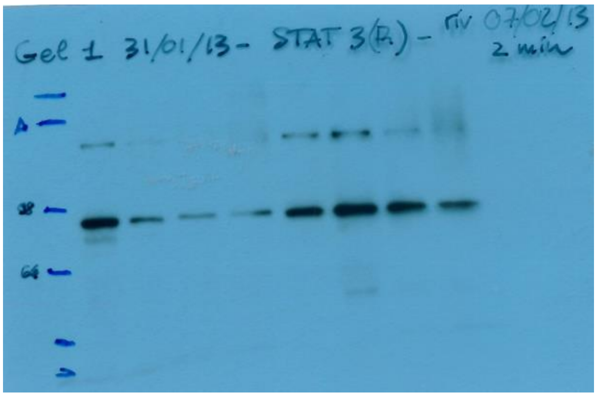

β-actin

KDa
148
98
64
50
43 →

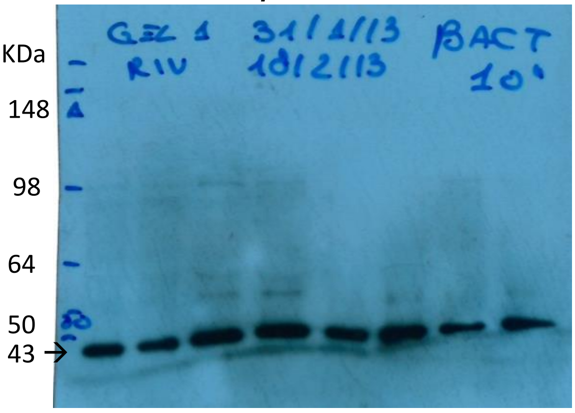

Figure 3D

24h CLL

PARP

KDa
148
116 kDa →
98
89 kDa →
64
50
36

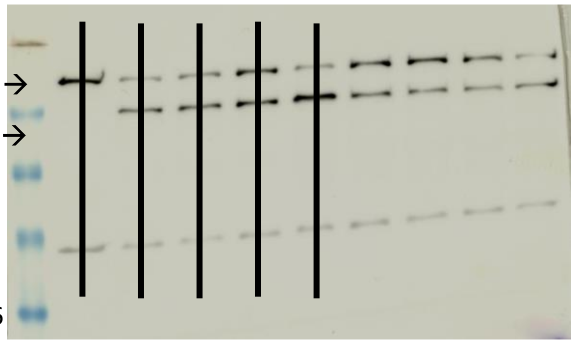

STAT3

P-STAT3 Tyr705

KDa
148
98
80 →
64
50
36

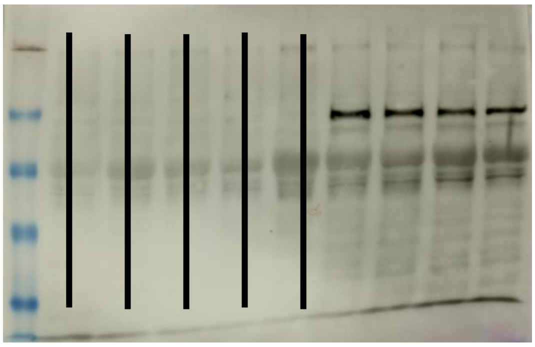

β-actin

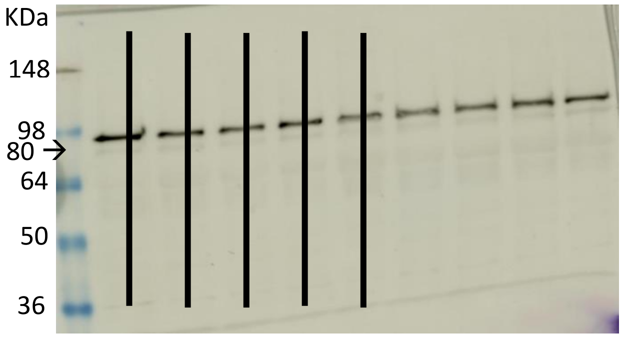

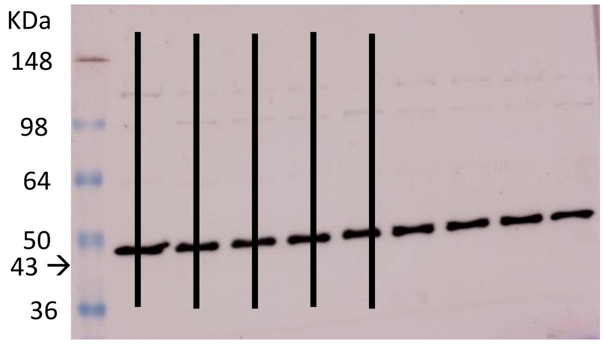

24h CLL + BMSCs

P-STAT3 Tyr705

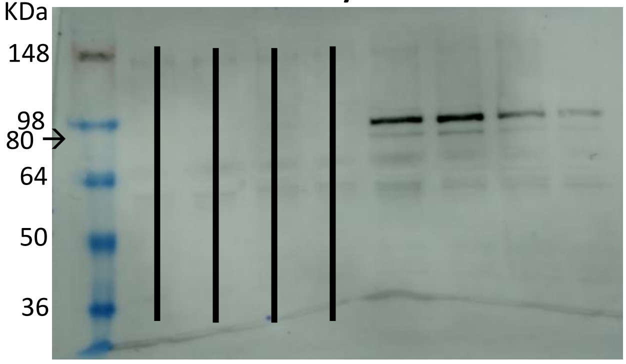

STAT3

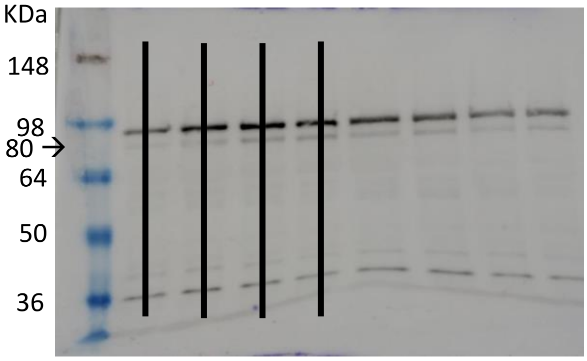

PARP and β-actin

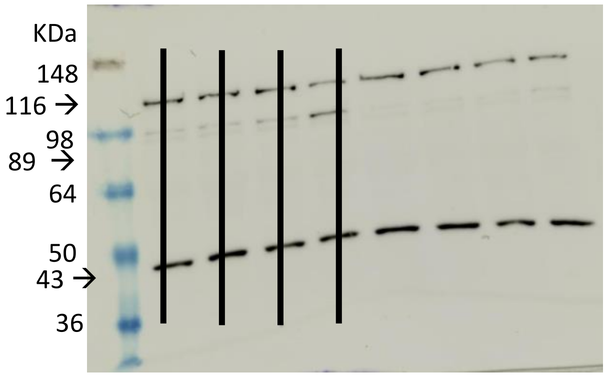

48h CLL

PARP

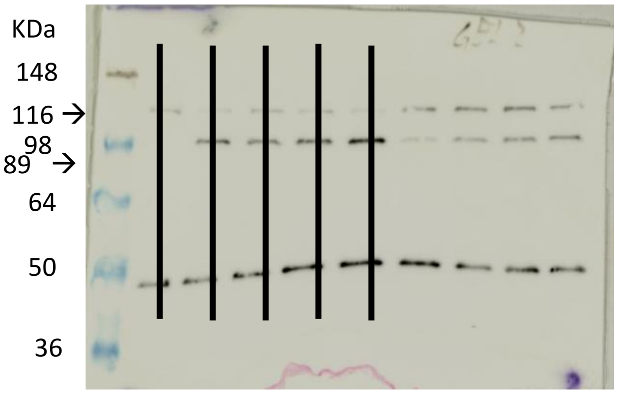

P-STAT3 Tyr705

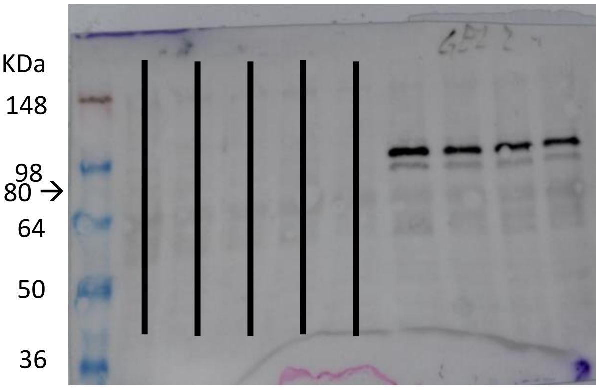

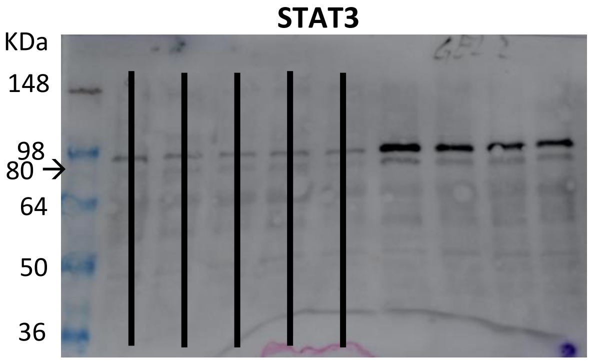

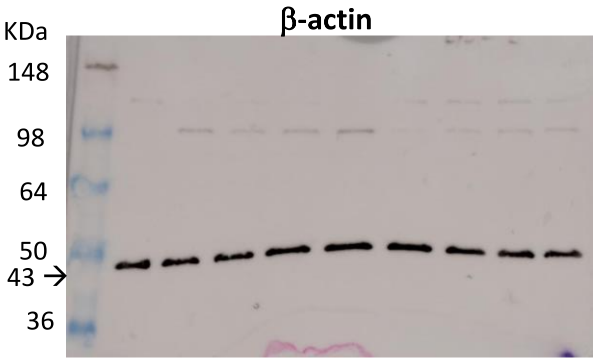

48h CLL + BMSCs

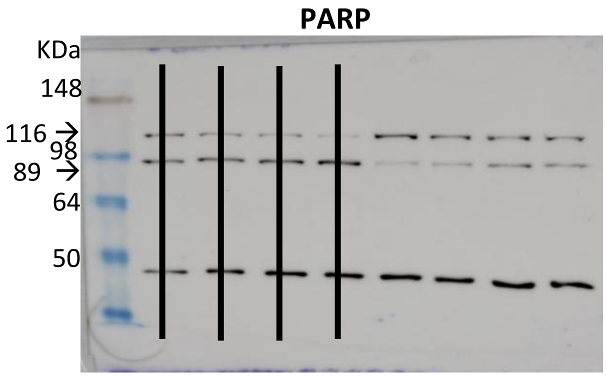

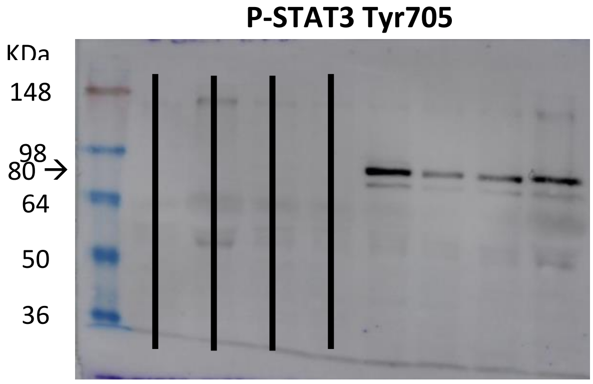

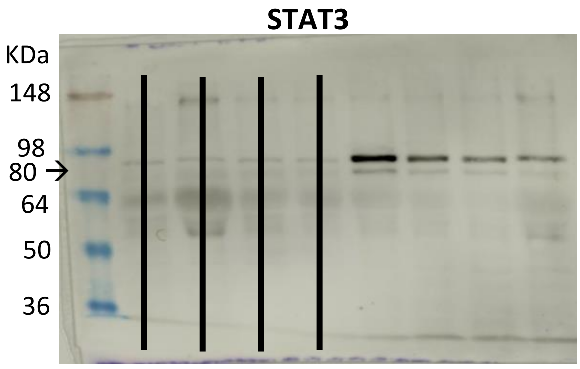

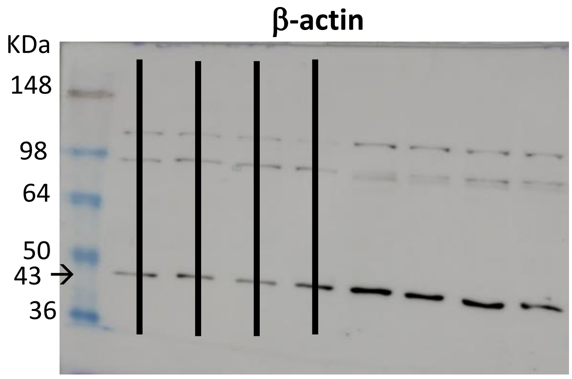

72h CLL

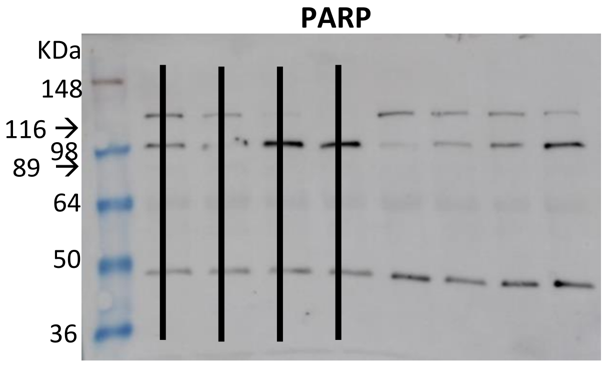

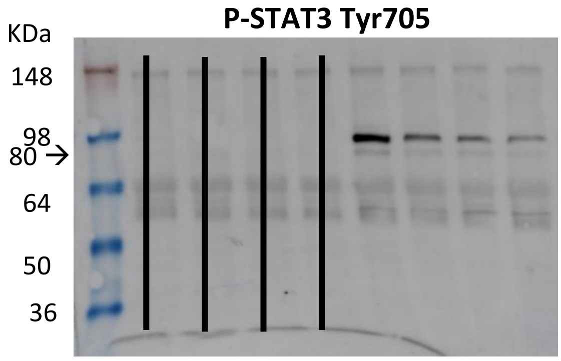

STAT3

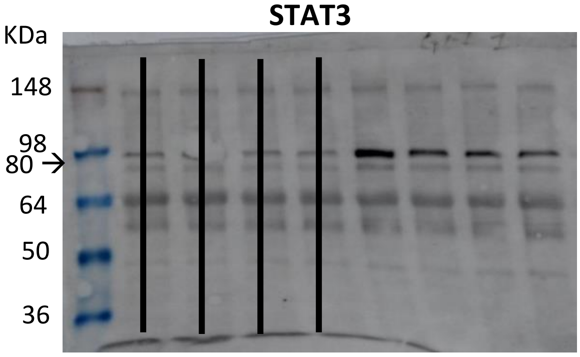

β-actin

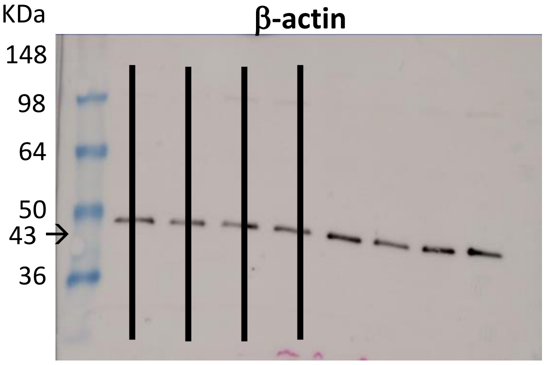

72h CLL + BMSCs

PARP

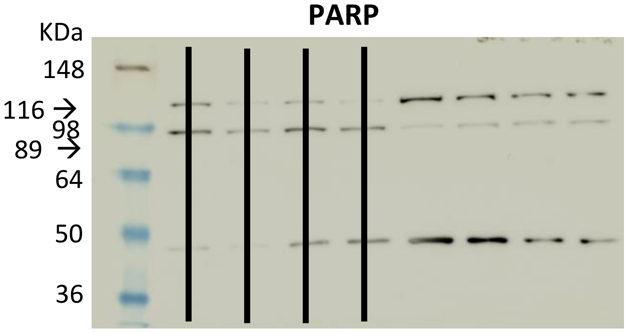

P-STAT3 Tyr705

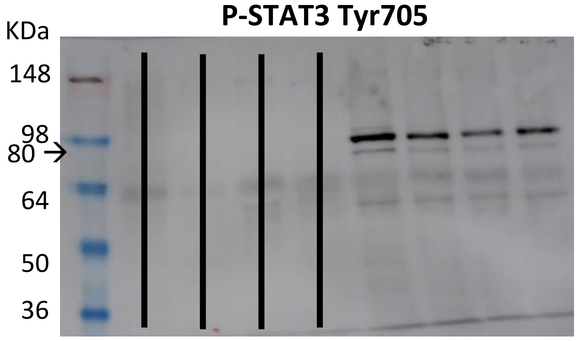

STAT3

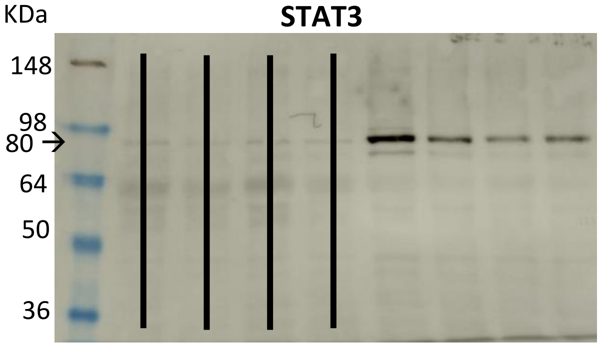

β-actin

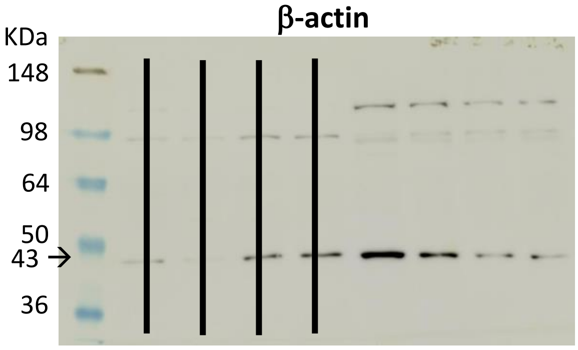

Figure 4A (left side)

P-STAT3 Tyr705

80 kDa →

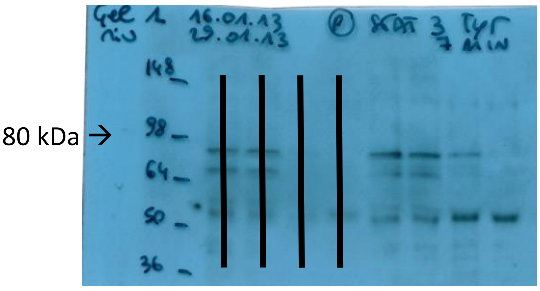

STAT3

80 kDa →

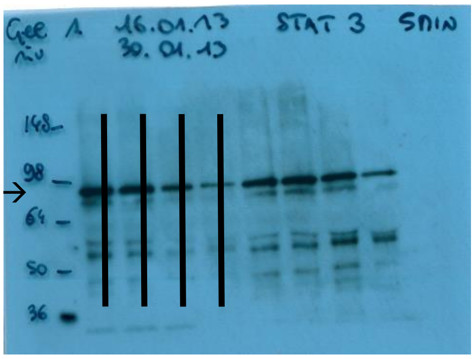

β-actin

KDa
148
98
64
50
43 →

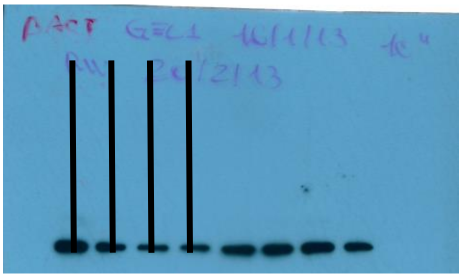

P-Lyn Tyr396

kDa
53/56 →

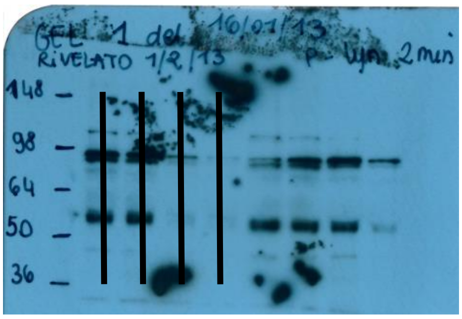

Lyn

kDa
53/56 →

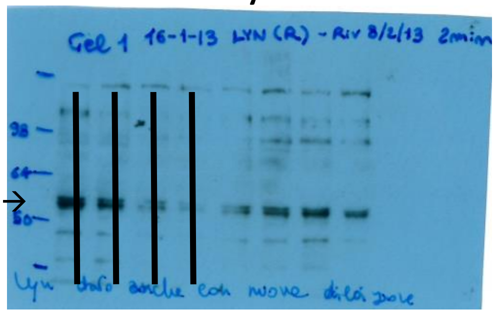

P-SHP-1 Ser591

68 kDa →

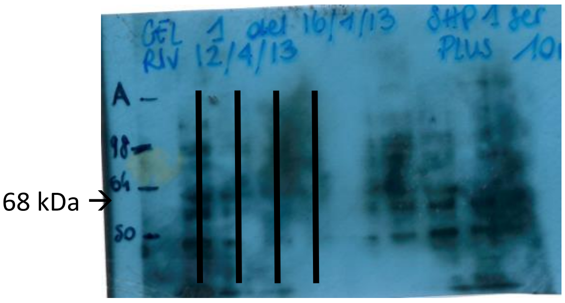

SHP-1

68 kDa →

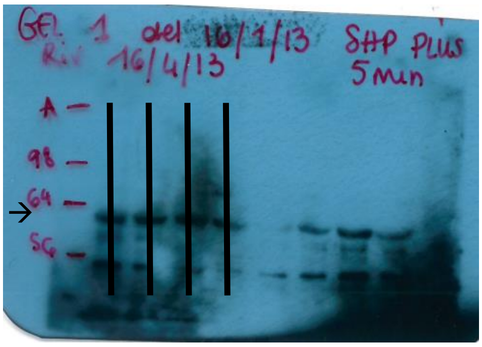

Figure 4A (right side)

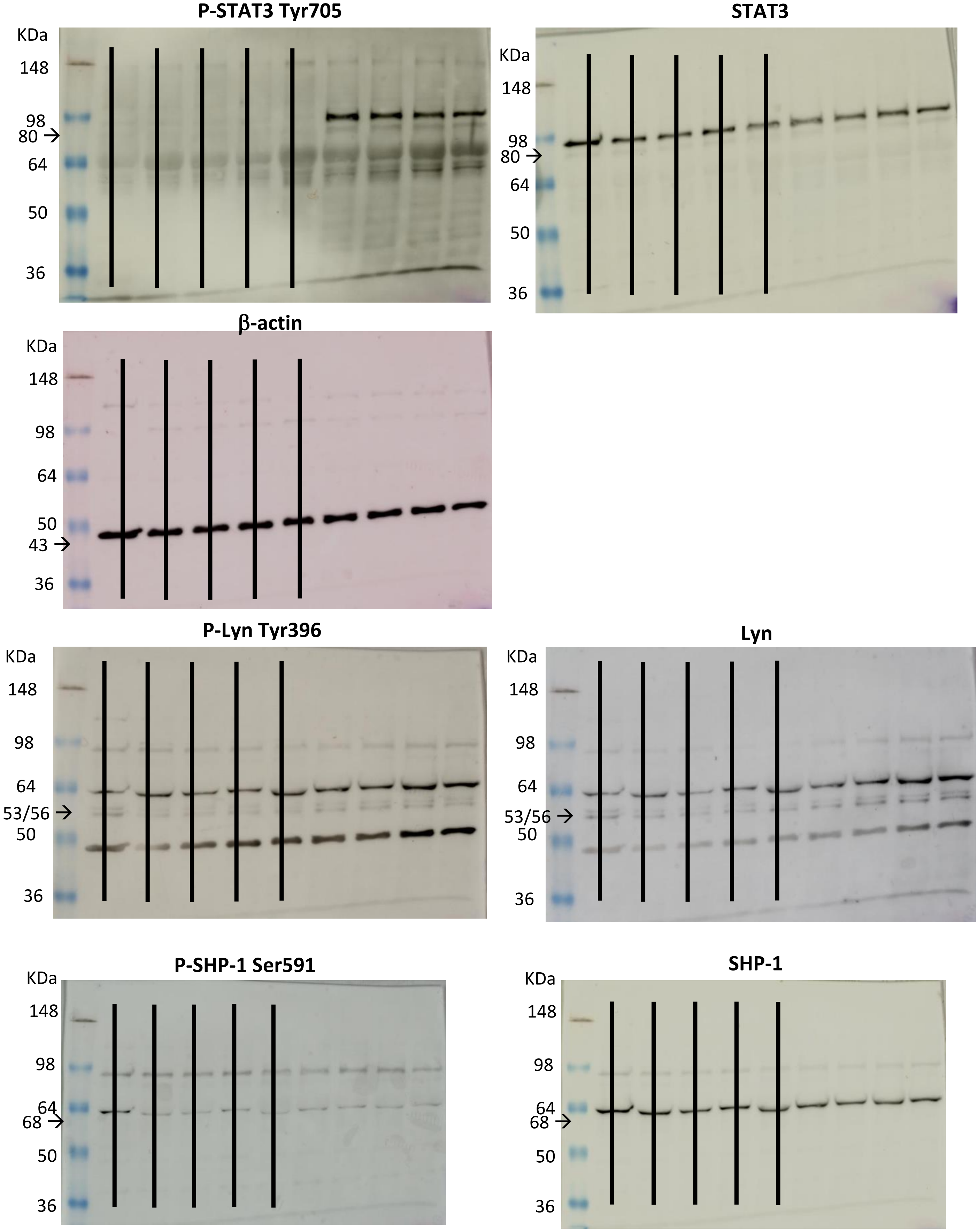

Figure 4B

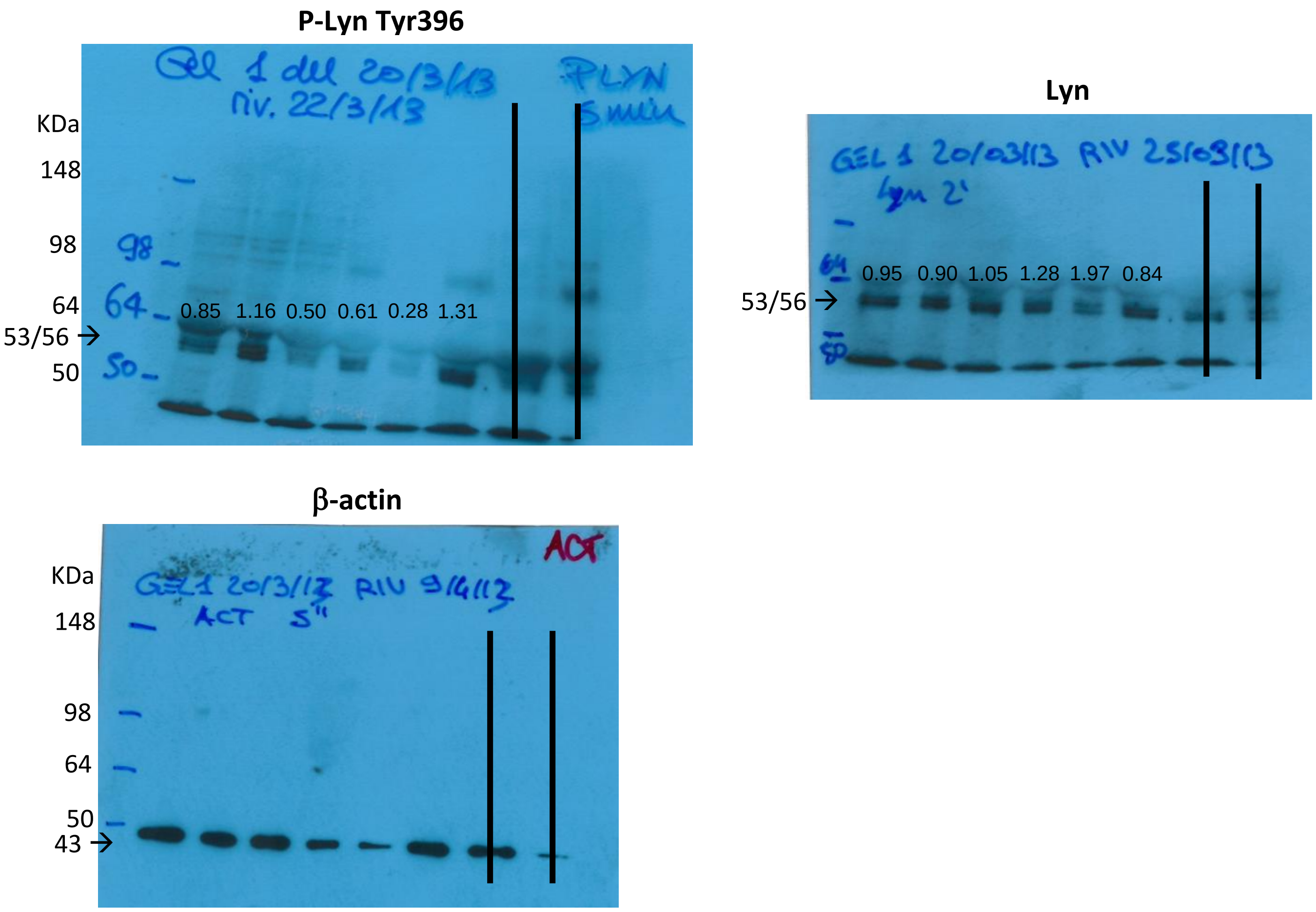
P-Lyn Tyr396
KDa
148
98
64
53/56
50
0.85 1.16 0.50 0.61 0.28 1.31
Lyn
53/56
0.95 0.90 1.05 1.28 1.97 0.84
β-actin
KDa
148
98
64
50
43

Figure S3A

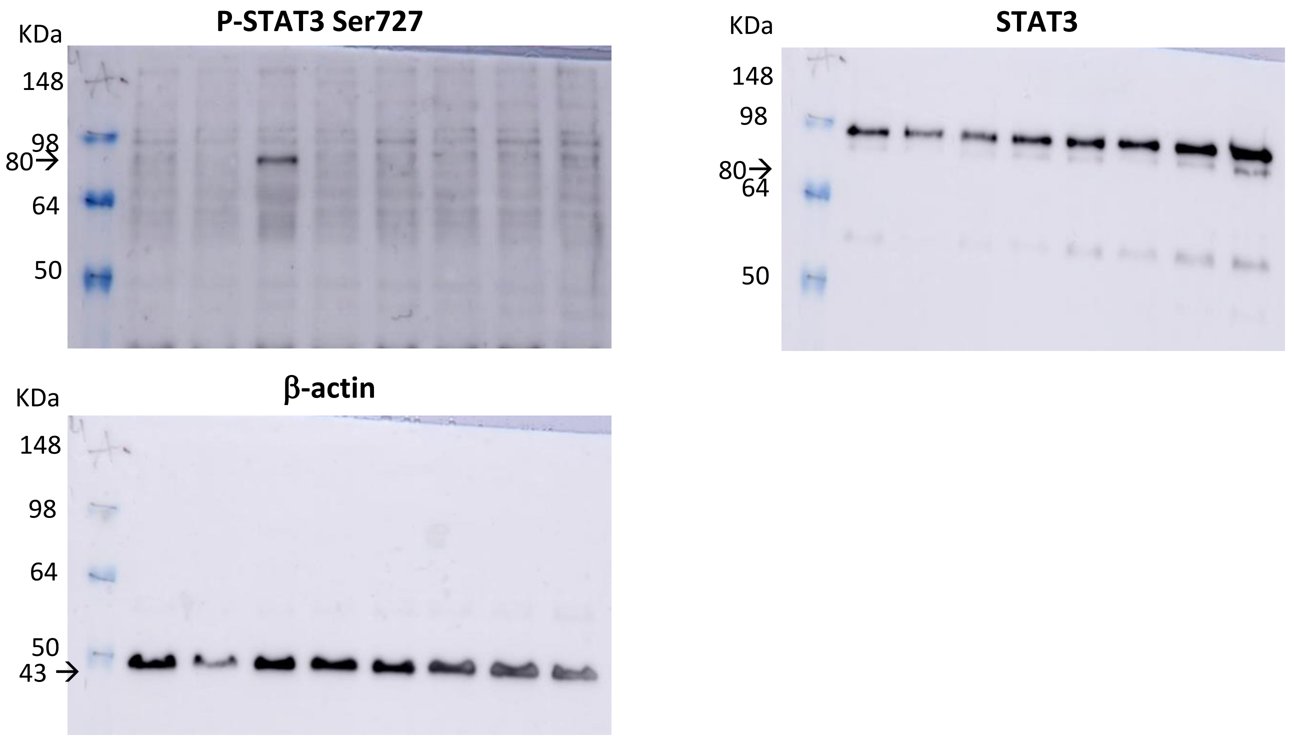

Figure S3B up

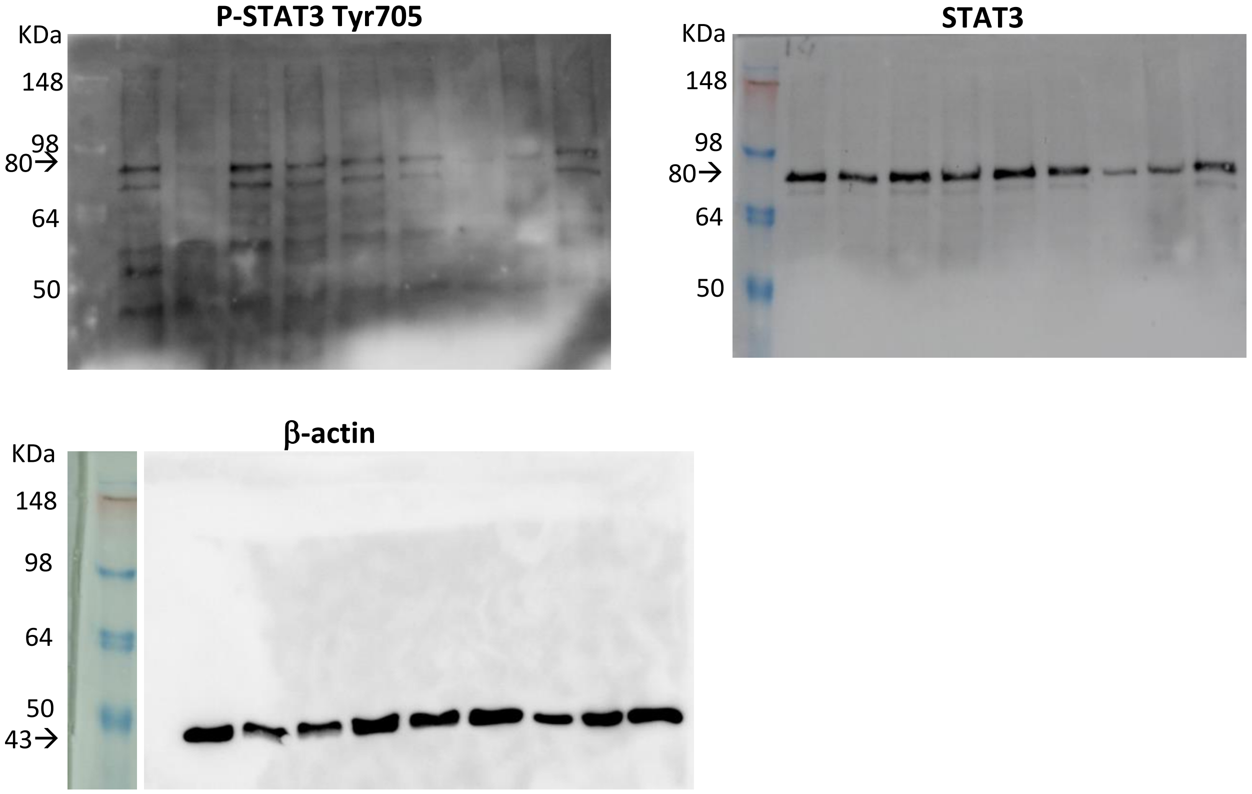

Figure S3B down

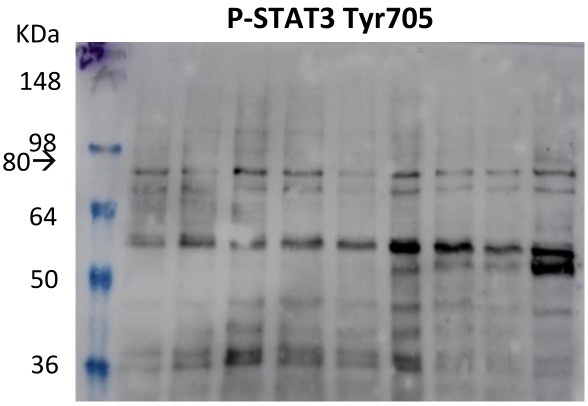

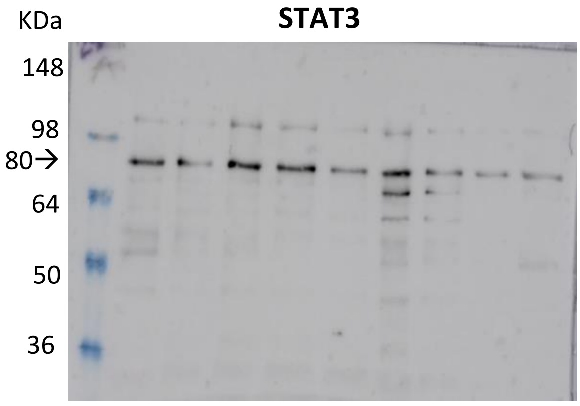

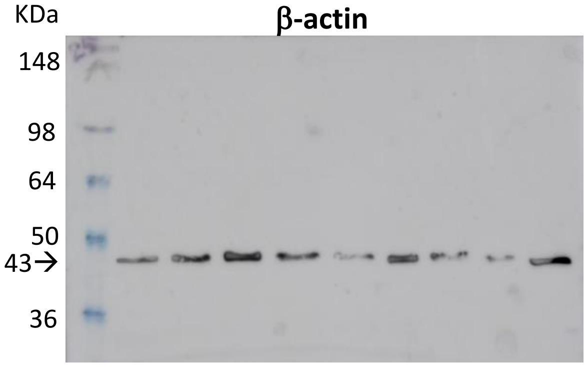

Figure S4A

Left – up

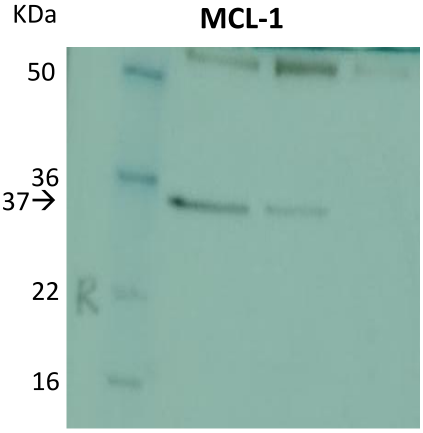

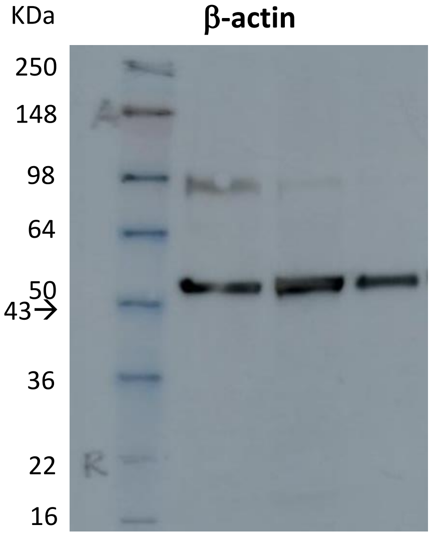

Left – down

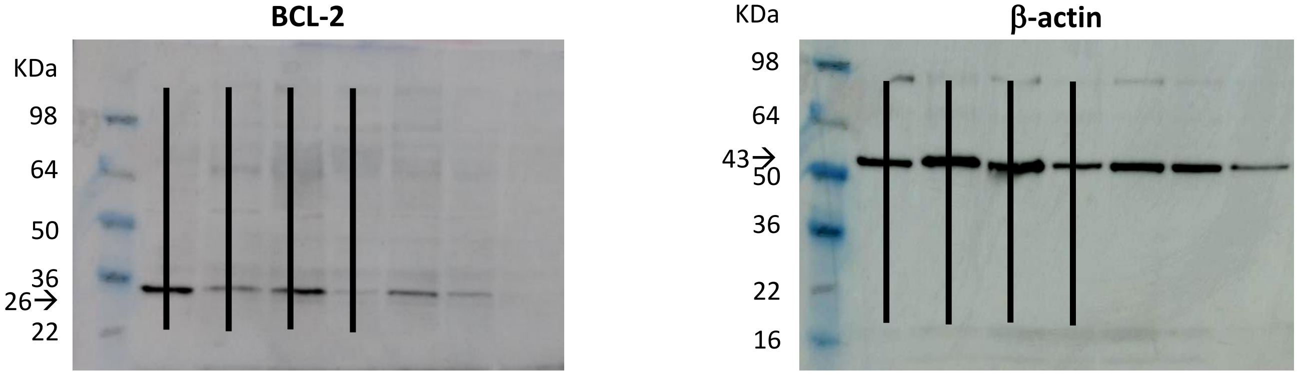

Right

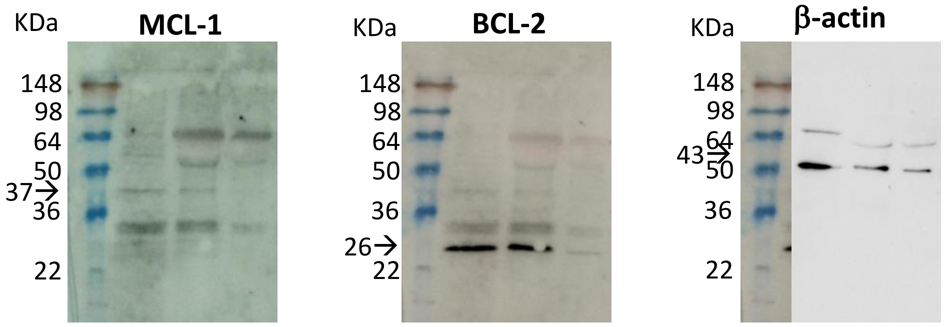

Supplementary Methods

STAT3 and JAK2 mRNA expression by real-time PCR analysis

Total cellular RNA from patient samples were extracted from $9x10^6$ leukemic cells using RNeasy Mini Kit (Qiagen; Hilden, Germany), according to the manufacturer's protocol and treated with DNase (Qiagen). First strand complementary DNA (cDNA) was generated from 1 μg total RNA using oligo-dT primer and the AMV reverse transcriptase (Reverse Transcription System, Promega Corporation; Madison, WI). Real-Time quantitative PCR amplifications reaction were carried out in ABI Prism 7000 sequence detection system (Applied Biosystems; Foster City, CA) in a 15 μl volume, as previously described [26]. To analyze the amplification results, delta-delta CT method was used. To distinguish specific amplicons from non-specific amplifications, a dissociation curve was generated. The primers used for GAPDH, STAT3 and JAK2 amplifications are: GAPDH F 5′-AATGGAAATCCCATCACCATCT-3′, GAPDH R 5′- CGCCCCACTTGATTTTGG-3′, STAT3 F 5′- AGGAGGAGGCATTCGGAAA-3′, STAT3 R 5′- AGCGCCTGGGTCAGCTT-3′, JAK2 F 5′-TTGGCAACAGACAAATGGA-3′ and JAK2 R 5′- CACTTTGTGGGAAATCTGCA-3′.
